# Generative adversarial networks for generating synthetic features for Wi-Fi signal quality

**DOI:** 10.1371/journal.pone.0260308

**Published:** 2021-11-23

**Authors:** Mauro Castelli, Luca Manzoni, Tatiane Espindola, Aleš Popovič, Andrea De Lorenzo

**Affiliations:** 1 Nova Information Management School (NOVA IMS), Universidade Nova de Lisboa, Lisboa, Portugal; 2 Dipartimento di Matematica e Geoscienze, Università degli Studi di Trieste, Trieste, Italy; 3 School of Economics and Business, University of Ljubljana, Ljubljana, Slovenija; 4 Dipartimento di Ingegneria e Architettura, Università degli Studi di Trieste, Trieste, Italy; Norfolk State University, UNITED STATES

## Abstract

Wireless networks are among the fundamental technologies used to connect people. Considering the constant advancements in the field, telecommunication operators must guarantee a high-quality service to keep their customer portfolio. To ensure this high-quality service, it is common to establish partnerships with specialized technology companies that deliver software services in order to monitor the networks and identify faults and respective solutions. A common barrier faced by these specialized companies is the lack of data to develop and test their products. This paper investigates the use of generative adversarial networks (GANs), which are state-of-the-art generative models, for generating synthetic telecommunication data related to Wi-Fi signal quality. We developed, trained, and compared two of the most used GAN architectures: the Vanilla GAN and the Wasserstein GAN (WGAN). Both models presented satisfactory results and were able to generate synthetic data similar to the real ones. In particular, the distribution of the synthetic data overlaps the distribution of the real data for all of the considered features. Moreover, the considered generative models can reproduce the same associations observed for the synthetic features. We chose the WGAN as the final model, but both models are suitable for addressing the problem at hand.

## 1 Introduction

Wireless networks are characterized by complex features, such as signal properties, channel quality, and frequency bands [1]. On the other hand, communication performance depends on several factors, including resource allocation, queue management, and congestion control. To handle this complex scenario, machine learning techniques have been widely used in the area of wireless networks [2]. Nowadays, with the increasing popularity and use of mobile devices, it is necessary to adapt and evolve the existing communication infrastructures to maximize user experience. In particular, wireless networks must support exploding traffic volumes and agile management of network resources and, for this reason, wireless networks are becoming more and more complex. This increasing complexity requires machine learning systems to analyze bigger datasets, and it has also highlighted the need for more intelligent and flexible algorithms [2]. To answer this call, recent years have witnessed the rising popularity of deep learning (DL) in the area of wireless networks [3]. By taking advantage of the existing hardware, better optimization algorithms, and the availability of a vast amount of data, DL fully exploits the power of artificial neural networks [4], and it is nowadays used in various settings and domains [5, 6]. In particular, DL models can accept as input several network parameters and can automatically discover complex hidden patterns that may successfully address complex tasks, such as interference alignment management [7] and signal detection and optimization [8]. As discussed in one research [3], compared to traditional machine learning techniques, DL provides several advantages in the context of wireless network applications, including higher prediction accuracy, and there is no need to pre-process input data. Due to these properties, DL-based systems have been used in the wireless network field to address several optimization problems at various layers. At the physical layer, DL was used for interference alignment [7], classifying the modulation modes [9], and designing efficient error correction codes [10]. At the data link layer, DL was used for resource allocation [11] and link quality evaluation [12]. At the routing layer, DL can optimize the routing path [13], whereas it was used to improve data compression [14] at the application layer. Altough the vast majority of the existing DL-based models are focused on the optimization of the functions related to the wireless network layers [3], DL models can be employed in other important areas. In particular, recent literature has focused on the security and privacy of wireless networks [15], thus showing the suitability of DL in addressing various tasks. In this paper, our focus is on the quality of a wireless network’s service. The work is motivated by the urgent need of service provider companies to promptly deal with technical problems in their network. These problems can affect the final user and cause failures in internet connectivity. Consequently, they will also affect the client’s satisfaction with the company, and, in the worst-case scenario, they will result in ubscription churn [16]. To ensure a high-quality service and customer satisfaction, service providers are continuously looking for solutions to avoid service interruption or, at least, solve possible connection troubles as soon as possible. To achieve this objective, telecommunication companies are working with partner companies that specialize in the development of software packages that can collect and analyze data from telecommunication networks. Collecting this data allows service providers to detect possible interruptions in the service before they affect the quality of service perceived by the final user—that is, the service provider’s subscriber. However, during software development, it is common for these partner companies to deal with the problem of data deficiency to perform the necessary tests, especially in countries where data protection laws are more rigorous. Due to this limitation, the simulation and subsequent use of fictitious datasets can provide a viable solution to the problem. Anyway, to guarantee the effectiveness of this approach, simulated data must be similar (ideally indistinguishable) to the real ones.

To achieve this goal, in this work we rely on generative adversarial networks (GANs) [17], which are state-of-the-art generative models, for the generation of synthetic data. These algorithms are based on the game theory and consider a framework with two neural networks that compete against each other. Althought this competitive network architecture has demonstrated impressive results when compared against previous generative models [18], there is an important limitation to consider: GANs are difficult to train [19] and, for this reason, researchers are investigating new techniques [20] to improve the original GAN architecture [21].

In this paper, we aim to assess the suitability of GANs for addressing a challenging task in the telecommunications domain. More specifically, the objective is to create synthetic features that can be used to measure the quality of Wi-Fi networks. To achieve this goal, we considered various GAN-based models, and, for each model, we analyzed (qualitatively and quantitatively) the resulting synthetic data to understand whether they can be distinguished from the real Wi-Fi network’s data. The best generative model obtained in this study is currently employed by a company that is partnered with one of the principal telecommunication providers in the Latin America area. In particular, the company uses the generative model for creating synthetic data to simulate and analyze varous scenarios that allow for continuous evaluation of the quality of the network’s signal. To the best of our knowledge, this is the first attempt to create a synthetic Wi-Fi network’s quality data through a GAN model. Moreover, altough GANs belong to the area of deep learning models, their application in the context of wireless networks is still in its infancy. A 2019 survey paper [3] on deep learning for wireless networks cited more than 100 papers using various DL-based models but generative models were not mentioned.

All in all, the main contributions of the paper, where GANs are used for the first time to generate synthetic Wi-Fi networks’ quality data, include the following:

- We propose the use of a GAN-based model to create quality data for a synthetic Wi-Fi network.
- We show, qualitatively and quantitatively, that an appropriate training process allows service providers to create a synthetic dataset that is almost indistinguishable from the real ones.
- We show that a machine learning classifier (i.e., random forests) poorly discriminates between real and synthetic data, thus corroborating the robustness of the GAN-based model.

The document is organized as follows. Section 2 provides an introduction to the Wi-Fi technology, explaining its main features, properties, and factors that can affect the quality of the signal. It also reviews basic concepts on the basic architecture of a GAN. Section 3 discusses related works where generative models have been used in the context of wireless networks. Section 4 details the two architectures considered in this paper and describes the dataset considered in the experimental phase. Section 5 discusses the results achieved by the considered models by highlighting their main differences and explaining how the best model was selected. Finally, Section 6 concludes the paper and summarizes the main achievements of this research.

## 2 Background

This section discusses basic concepts on Wi-Fi networks and GANs that should allow the reader to understand the subsequent parts of the paper.

### 2.1 Wi-Fi concepts

Developed to replace Ethernet cables, these days Wi-Fi is a very popular technology that is applied almost everywhere and provides fast and efficient interconnectivity between devices.

Wi-Fi networks can use two frequency bands: 2.4 GHz or 5 GHz. Each of these frequency bands has several channels, which are considered as smaller bands on which wireless devices can operate on [1]. Two important components in a Wi-Fi network are the modem and the router. The modem is the equipment required to access the internet once it connects devices with an internet service provider, or ISP. The router is the interface between the modem and the wirelessly connected devices. Nowadays, it is common to find devices that combine both functions. A generic term that referring to all kinds of communication equipment physically located at the subscriber’s home and connected with a carrier’s telecommunication circuit is CPE (i.e., customer premises equipment or customer provided equipment) [22]. Nowadays, many CPEs are dual-band and give the user the possibility of choosing between the two frequency bands: 2.4 GHz or 5 GHz. The main difference between these two frequency bands is the range and bandwidth that they provide. A band of 2.4 GHz has a bigger Wi-Fi coverage, whereas a 5 GHz band has a faster speed.

A very important characteristic of a Wi-Fi network is signal strength. It can be understood as the wireless signal power level received by the user and is dependent on the router transmit power, the frequency used, the distance traveled by the signal, and so on. This value is a key factor in the activities for which the network can be used. A stronger signal strength results in more reliable connections and higher speeds. Several factors can impact the strength of a Wi-Fi network’s signal, causing it to vary between devices connected in the same CPE (e.g., router location, distance of the device from the router, walls and floors, interference from other devices).

To ensure good performance in a wireless environment, wireless devices must distinguish between the received signals that are legitimate information and listen for background signals that should be ignored. In this context, another measurement regarding the quality of the Wi-Fi network is the signal-to-noise Ratio (SNR), which is the difference between the received signal and the background noise level [23].

### 2.2 Generative adversarial networks

A GAN is a DL-based [24] generative model that was introduced by Ian Goodfellow and other researchers at the University of Montreal in 2014 [17]. The term “adversarial” in used the algorithm name because its architecture consists of a system with two neural networks [25] that compete against each other and, through this competitive process, can generate synthetic instances from scratch [17].

The first neural network is called the generator, and its function is to generate synthetic data instances. The second neural network is called the discriminator, which attempts to distinguish between samples from the training data and samples drawn from the generator. In other words, the generator is trained to output instances as close as possible to the real ones and, therefore, fool the discriminator. On the other hand, the discriminator is trained to become better in determining which data are real and which are synthetic [17]. GANs are based on game theory, so the basic idea is to set up a game between two players or adversaries [26].

As the first step of this process, the generator generates a random batch of samples. Subsequently, these samples are provided to the discriminator jointly with another batch of real samples, and the discriminator is trained to identify the differences between them. Once these differences are identified, the discriminator will provide feedback to the generator. The generator relies on this feedback for improving the generative process, thus creating more realistic instances [17]. Both neural networks are trained in alternating steps. At each step, the discriminator will get better at distinguishing between real and fake instances and the generator will improve in generating realist samples that can trick the discriminator. In this sense, the two models are considered adversaries, once they are competing against each other [26].

**Fig 1:**
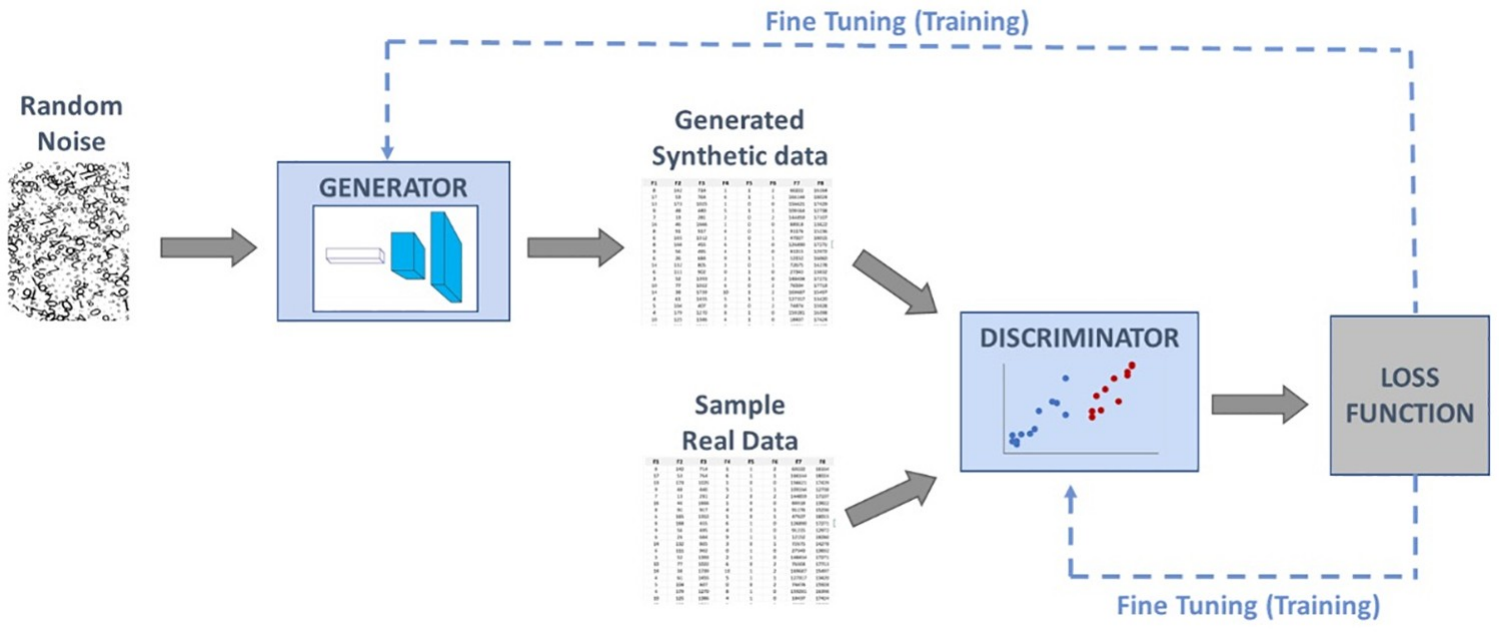
Representation of the general flow of a standard GAN architecture.

Fig 1 presents an illustration of this general flow of a GAN.

The final objective is to reach a situation in which the generator can generate perfect replicas of the samples in the input domain and the discriminator cannot distinguish between real and synthetic data. When the two models are sufficiently trained and cannot improve anymore, it is common to say that the network achieved the Nash equilibrium. Unfortunately, finding the Nash equilibrium is a complex task, that is more difficult than optimizing a unique objective function [17]. In particular, as the algorithm considers two networks simultaneously, as competitors, improvements in one model come at the expense of the other. Another problem happens when the discriminator becomes too successful in distinguishing between real and fake instances so that the generator gradient vanishes and learns extremely slowly or next to nothing. This problem is known as a vanishing gradient or a loss saturation [19, 27]. Furthermore, real data distributions are highly complex and multimodal. In other words, the data distribution has a lot of peaks that represent a concentration of similar data samples. One drawback in GANs occurs when the output produced by the generator is concentrated in a limited set of these peaks. This problem is called mode collapse and limits the diversity in the generated samples [28]. To overcome these limitations, more advanced GAN architectures were defined, including the Wasserstein GAN architecture described in section 4.

## 3 Related work

This section examines the recent major contributions where GAN-based models have been used in practical applications. In particular, the section focuses on the context of Wi-Fi networks.

Although recent years have witnessed the application of several deep learning models in this area, we focus our attention on GAN-based models, and we refer the reader to the existing literature [3, 29] for a survey of deep learning models in mobile and Wi-Fi networks. Despite the rising interest in generative models, GANs have been proposed only recently in the field of wireless networks. The main reason is that GAN-based architectures are mainly used in computer vision, and the effort of the scientific community is focusing on improving the quality of the generated images [30]. One of the applications of GANs in wireless communication is for modeling wireless channel response. Channel modeling is a fundamental task for the accurate design and performance evaluation of a network. Although existing works in designing or learning new modulation schemes have focused on using simplified analytic channel [31, 32], O’Shea *et al*. [33] relied on GANs to approximate wireless channel responses to more accurately reflect the probability distribution functions (PDFs) of stochastic channel behaviors. To achieve their objective, they considered variational GANs [34] that relying on appropriate loss functions can efficiently capture these stochastic behaviors. Experimental results demonstrated the suitability of the proposed approach for channel modeling. Moreover, the authors analyzed the performance of a simple GAN (without the variational layer) and highlighted the importance of using the variational sampling layer for achieving better performance. In the same vein, Ye *et al*. [35] proposed an end-to-end wireless communication system using deep neural networks. In their system, they proposed using a conditional GAN to model channel effects in a data-driven way, where the received signal corresponding to the pilot symbols is added as a part of the conditioning information of the GAN. Experimental results showed the effectiveness of the system with various channels, and the authors highlighted the possibility of building data-driven deep neural networks for end-to-end communication systems. Compared to one research [33], the work of Ye *et al*. [35] can be applied to more realistic fading channels, thus corroborating the suitability of GAN-based architectures for channel modeling. Balevi and Andrews [36] focused their study on the wideband channel estimation, and they presented the use of GANs for this purpose. In more detail, they designed a GAN to learn to produce channel samples according to its distribution and then use this knowledge as a priori information to estimate the actual current channel by optimizing the network’s input vector in light of the current received signal. Thus, this approach is different from using GANs for channel modeling [33, 35, 37]. A similar approach is used in other works of the same authors [38, 39], in which GANs are used for high-dimensional channels estimation when considering various wireless networks’ parameters. More recently, GANs were combined with deep reinforcement learning (deep-RL) to create a framework for providing model-free resource allocation for ultra-reliable, low-latency communication in the downlink of a wireless network [40]. The idea is to pre-train the deep-RL framework with both real and synthetic data, thus creating a deep-RL system that experiences a broad range of network conditions. Experimental results have shown that the use of GANs contributed to the high reliability of the system. Thus, synthetic data produced by GANs can be effectively exploited for improving the overall communication system. Beyond the aforementioned works, where GANs were used at the physical layer for channels modeling, recent contributions are proposing the use of GANs for specific Wi-Fi related applications, mainly related to security. In one research [41], the authors presented a GAN-based spoofing attack to generate synthetic wireless signals that cannot be statistically distinguished from intended transmissions. The idea is the following: the adversary transmitter trains a deep neural network to generate the best spoofing signals and fool the best defense trained as another deep neural network at the adversary receiver. Experimental results showed that the GAN-based spoofing attack increases the attack success probability from 76.2% to 100% depending on the number of antennas used. The results demonstrated the ability of GANs in generating synthetic (and realistic) data and highlighted the need for developing defense mechanisms to detect and mitigate these GAN-based spoofing attacks. In the same vein, Lin *et al*. [42] proposed a GAN-based framework to generate the adversarial malicious traffic records aiming to attack intrusion detection systems (IDSs) by deceiving and evading the detection. Experimental results demonstrated the effectiveness of the method and showed that the vast majority of the attacks are not discovered by the IDSs. On the other hand, Hang and Lei [43] proposed a GAN-based system for improving the performance of IDSs. The authors observed that IDSs work as a binary classifier, where the two classes correspond to normal samples and anomalies. The main issue is that there is a significant class imbalance, and that the number of abnormal samples (i.e., anomalies) is significantly lower than that of the normal ones. This class imbalance problem constrains the performance of IDSs and results in low robustness to unknown anomalies. Thus, the author presented a GAN-based system to address the class imbalance problem by creating synthetic samples corresponding to anomalies. Experimental results demonstrated the suitability of the proposed method. In particular, by generating synthetic data to obtain a balanced dataset of normal and abnormal samples, the performance of the IDS improved compared to the use of well-known oversampling techniques. In this paper, we present a different application of GANs. Our objective is to generate synthetic features related to Wi-Fi networks to improve the quality of service perceived by the user. By generating synthetic data, we expect that we can cover a broad range of situations representing possible connection problems and service interruptions. The generation of this synthetic data would allow software companies partners of service providers to better analyze and understand the situations that lead to poor service.

From a more general point of view, generative models have found considerable success in many fields of computer vision, such as semantic image synthesis [44], image-to-image translation [45] and super resolution [46]. Remarkable results in this field have been achieved thanks to the introduction of progressive GANs: the authors of [47] developed a GAN architecture that progressively grows during the training. The network begins to generate images in low resolutions and, step by step, it duplicates its size until it reaches high-quality resolutions. The greatest advantage of this technique is that it makes it possible to reduce the time required in training the GAN to generate high-resolution images. Moreover, this architecture helps the network to learn gradually as the problem is introduced in a simplified version (the low resolution image), which gradually becomes more complex. Recently, in the field of image generation, StyleGAN [48] and its improved version, StyleGAN2 [49], have enjoyed considerable success. Starting from their previous work about progressive GANs, the authors of StyleGAN noticed how the various layers in the growing architecture are capable of controlling different visual features of the generated image. This insight prompted the authors to introduce two new components: the Mapping Network and the Style Module (AdaIN [50]). The former is a network that encodes a sample from the latent space into a vector *w* whose different elements control different visual features and can feed the various layers of the GAN. The latter is a module that helps to transfer the visual features from the *w* vector into the generator. Its operation can be broadly divided into two parts: one for normalizing the image generated from the previous layers and one for entering information relating to the image style. In particular, the second one is achieved by transforming *w* into scale and bias values trough another fully-connected layer. One of the most recent improvements of StyleGAN is the introduction of adaptative discriminator augmentation (ADA) [51]. The goal of that paper is to demonstrate how a wide range of fine-tuned augmentations can improve the training preventing the discriminator to overfit. This mechanism was introduced in order to cope with problems where the amount of data is limited. It is important to note that all of these techniques, while extremely successful, are not applicable to the study presented in this article due to the nature of the data involved. These are not in the form of a time series or a signal, but are vectors of statistics without any spatial relationships between them. Therefore, any convolutional approach loses sense in this scenario.

Although computer vision is the application field with the greatest use of GANs, these have also been successfully used in the generation of synthetic data. One example is the generation of synthetic tabular data with tableGAN [52]. The idea behind this paper is to generate surrogate data that can be usable for training classifiers when real data are scarce or unavailable for privacy reasons. Privacy preservation is central to this work; therefore the authors have focused on developing a loss function that takes into account the anonymization of the generated data: when the loss is higher, the generated data are more anonymous. A similar approach has been proposed with TGAN [53], which evaluated the generative process on actual medical records. In particular, this work introduced the usage of long-short term Memory (LSTM) cells into the generator architecture. This choice was made in order to exploit the spatial relationships between the features of the problem. The assessment of this proposal has been done on three real-world medical datasets. Choi *et al*. [54] proposed another usage of GANs, medGAN, for generating synthetic medical data. The application is tailored to the generation of electronic health records (EHRs) and is particularly focused on the removal of sensible information that can emerge from the record used as training examples.

## 4 Experimental settings

This section presents the GAN architectures considered in the experimental phase and describes the data used in this work.

### 4.1 GAN architectures

This section presents the GAN architectures considered in this study. The first one is the vanilla GAN, the original GAN architecture proposed in the literature [17]. The second architecture is called WGAN [55] and considers a different loss function that compares how close the distribution of the generated dataset is from the distribution of the real data. GANs try to replicate a probability distribution. Thus, they should use a loss function that reflects the distance between the distribution of the real data and the distribution of the generated fake instances.

#### 4.1.1 Vanilla GAN

In the vanilla GAN, once the model has two neural networks, two loss functions are considered: one for the generator and one for the discriminator. However, the two loss functions derive from a single measure of distance between the two probability distributions being compared: the one outputted by the generator model and the one from the real data [17]. The loss function used in the literature [17] was the minmax loss, which is based on the cross-entropy. As the name suggests, it works like a min-max game where the generator tries to minimize it (generating synthetic samples that are very similar to the real ones), whereas the discriminator aims at maximizing it (by distinguishing between fake and real instances).

The formula of this loss function is given in Eq 1:

$$\begin{array}{c} \begin{array}{c} \underset{G}{\text{min}}\;\underset{D}{\text{max}}\;V\left(D,G\right)=E_{x∼\;P_{data(x)}}\left[logD\left(x\right)\right]\\ +E_{z∼\;P_{z(z)}}\left[log\left(1-D\left(G\left(z\right)\right)\right)\right] \end{array} \end{array}$$ (1)

where *E*_*x*_ is the expected value over all real data instances; *D*(*x*) is the discriminator’s estimate of the probability that a real data instance *x* is real; *E*_*z*_ is the expected value over all of the generated instances *G*(*z*); *G*(*z*) is the generator’s output when given a noise vector *z*; and *D*(*G*(*z*)) is the discriminator’s estimate of the probability that a generated instance *G*(*z*) is real.

As the formula shows, two terms are used to measure the ability of the discriminator in correctly recognizing the samples. The first one, $E_{x∼\;P_{data(x)}}\left[logD\left(x\right)\right]$ measures its capacity in recognizing the real instances, while the second one, $E_{z∼\;P_{z(z)}}\left[log\left(1-D\left(G\left(z\right)\right)\right)\right]$, measures its capacity in recognizing the generated ones.

Another important point is that the generator model only affects the term related to the generated data. Thus, during the training of the generator, the term that considers the real data is dropped. The updates in the discriminator parameters are based on the values of the loss function considering both real and generated samples, whereas the updates in the generator parameters are based on the values of the loss function considering only the generated data.

#### 4.1.2 Wasserstein GAN

In 2017, Arjovsky and coauthors [19, 55] demonstrated that the loss function proposed in the original GANs paper can fail in some cases, thus resulting in poor performance of the network. To overcome this limitation, they proposed the use of the Wasserstein-1 distance metric, also known as the Earth Mover or EM distance. Although the entropy-based loss can be considered as a measure of how accurately the discriminator classifies real and generated data, the Wasserstein metric looks at the distribution of each variable in the real and generated samples and outputs a number that determines how far apart the distributions are from each other [55]. The Wasserstein metric not only evaluates if an instance is real or not but provides “criticism” on how far the generated data are from the real dataset. For this reason, the “discriminator network” in a WGAN architecture is frequently referred to as the “critic network” [55]. For each instance, the critic network outputs a score that quantifies how far the generated data are from the real distribution. The loss function of a WGAN can be expressed in a simple form, based on Eq 2. For more details on the Wasserstein metric, the reader is referred to the Appendix A of [55].

$$\begin{array}{c} WGAN_{loss}=f\left(x\right)-f\left(G\left(z\right)\right) \end{array}$$ (2)

where *f*(*x*) is the critic’s output for a real instance; *G*(*z*) is the generator’s output for a noise *z*; and *f*(*G*(*z*)) is the critic’s output for a generated instance.

As it is possible to notice, the loss can be implemented by calculating the average predicted score (i.e., the critic’s output for the considered minibatch) across real and fake data and then multiplying these averages by 1 and -1, respectively. This has the desired effect of driving the scores for real and generated data apart. The critic network tries to maximize this function. That is, it tries to maximize the difference between the critic’s output for real and synthetic instances [55]. The generator, as in the minmax function, only affects the term related to the generated data, *f*(*G*(*z*)) The use of the EM distance has shown improvements in the stability of learning and in getting rid of some convergence problems that are more common when considering the original GAN architecture. Empirically, it was also observed that the WGAN value function appears to correlate with generated data quality, which provides meaningful learning curves that are very useful for hyperparameter explorations [55].

### 4.2 Data

This section describes the set of real data used for generating synthetic data concerning Wi-Fi networks. Concerning the experimental phase, all of the GANs models were developed and trained using Keras. The training process was carried out on a computer with a CPU Intel Core i7 with 16GB of RAM.

The dataset used in this paper belongs to a Latin American telecommunications company and contains the data collected during April 2020 See S1 Dataset. Given the restrictions concerning data protection, it is not possible to describe all of the features in detail. Thus, this section will present an overview of the whole dataset and some additional information about the most important features. The dataset is composed of key performance indicators (KPIs), regarding 1,595 devices connected to 225 distinct CPEs. All of these indicators are calculated considering the monitored point vision, which considers the connected device, the CPE, and the CPE’s access point jointly. Therefore, if a specific device was connected to two different CPEs during the considered period, it was considered as two distinct monitored points. In addition to the calculated KPIs, the dataset also includes features with information concerning some attributes of these monitored points, such as router model and manufacturer, radio operating frequency, and so on.

**Fig 2:**
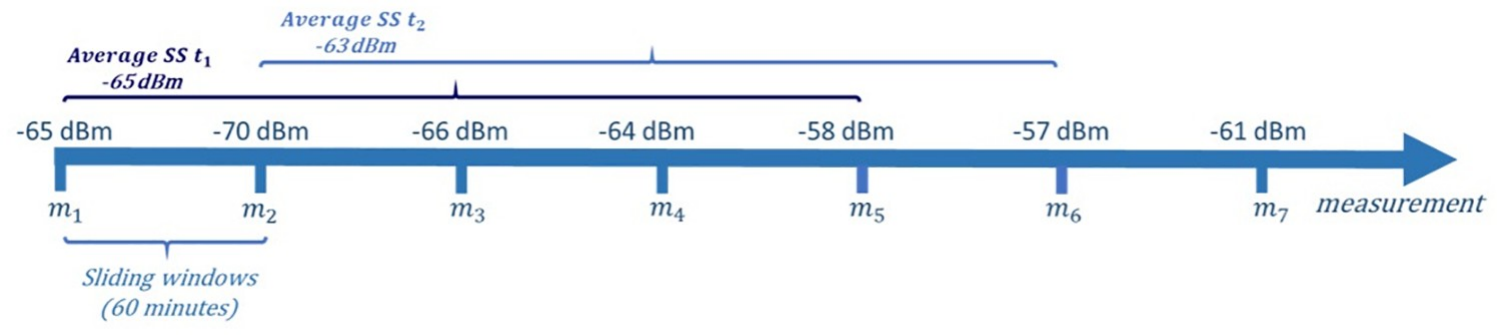
Example of KPIs computation.

Regarding the KPIs themselves, some examples that can be indicated are statistics regarding the signal strength and SNR (minimum, maximum, average, and variance during the considered period), the number of times the device changed the channel and the number of authenticated failures. Furthermore, all of the dataset’s KPIs were computed considering sliding windows of 5 units, each one with a collection interval of 60 minutes. As an example, if the KPI “average signal strength” for a specific device *d* in the time *t* is -65 dBm, that means the average of all of the last 5 measurements for that device, performed with intervals of 60 minutes, was -65 dBm (Fig 2). The same logic is used to measure all of the remaining KPIs.

After the preliminary analysis, the final dataset ended up with seven features that characterized the Wi-Fi signal. Four of them are the statistics regarding the signal strength in every time unit: minimum, average, maximum, and variance; one feature indicating the number of times the device was connected; and, finally, two additional features about the CPE: radiofrequency and manufacturer.

**Fig 3:**
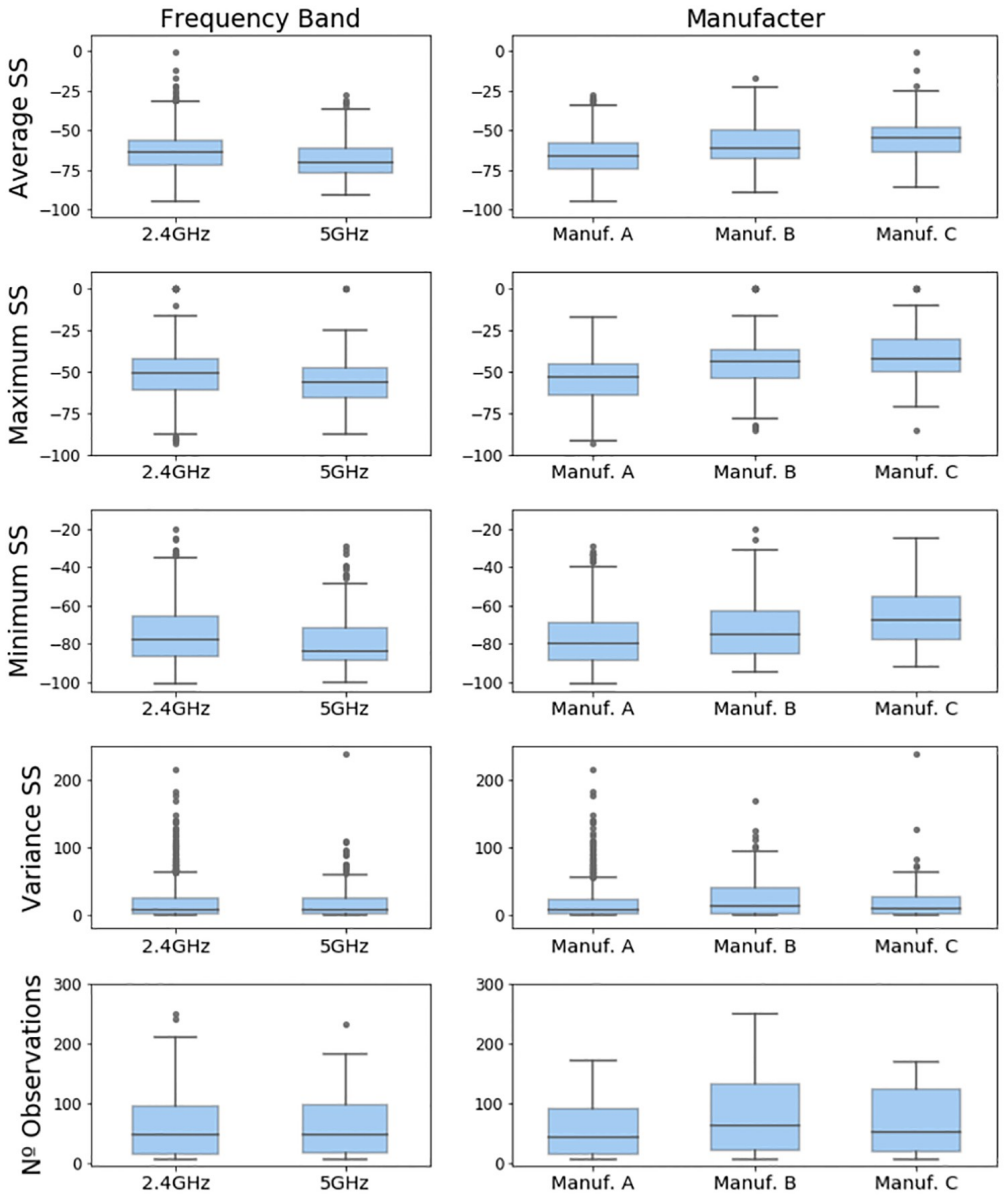
Distribution of the features of the considered dataset for each frequency band and manufacturer.

The boxplots in Fig 3 summarize the distribution of these features for the considered frequency bands and manufacturers.

## 5 Results

This section discusses the main results for both of the architectures considered. It presents and analyzes the simulated datasets outputted for each model, and compares them with the real data. The discussion of the results is divided into three section. In the first one, the results obtained with the vanilla GAN are presented. The second section examines the results obtained with the WGAN architecture. Finally, the best models produced by each architecture are compared against each other by relying on the use of random forests. After this analysis, a final model was selected. This model is currently used within the telecommunication company to generate synthetic data based on the real ones. These synthetic observations are used by the partner company (that cannot access and use real data) as the input for the software that controls the KPIs associated with the quality of the Wi-Fi networks. In this way, if the KPIs are not reaching some predefined quality thresholds, the telecommunications company may quickly take all of the necessary actions to restore itself as a high-quality service.

### 5.1 Vanilla GAN

For the vanilla GAN, the training process started considering a simple model topology with just one hidden layer for both of the neural networks. A z-score normalization was applied to the considered features in order to remove any bias that is the result of considering the unnormalized data. The hyperparameter’s values considered in the first model are listed below: Generator Model:

- Distribution of the noise vector *z*: Normal (0,1)
- Dimension of the noise vector *z*: 100
- Number of layers: 3—the input layer, one hidden layer, and the output layer
- Number of neurons in each layer: input layer—100; hidden layer—200; output layer—5
- Activation functions: input and hidden layers—LeakyReLU; output layer—Hyperbolic tangent

Discriminator Model:

- Number of layers: 3—the input layer, one hidden layer, and the output layer
- Number of neurons in each layer: input layer—200; hidden layer—100; output layer—1
- Activation functions: input and hidden layers—LeakyReLU; output layer—Sigmoid

Composite model and train process:

- Optimizer: Adam with a learning rate of 0.0002 and a momentum of 0.5
- Batch size: 120
- Number of epochs (stopping condition): 3000
- Number of updates of the discriminator per generator update: 1

It is worth noticing that the number of neurons in the output layers is fixed for both networks. For the generator model, this number must be equal to the number of features in the dataset once the generator outputs a synthetic instance. Concerning the discriminator model, there is one neuron that outputs a value between 0 and 1. This value can be interpreted as a probability and is used for classifying the input as real or synthetic. The Adam optimizer [56] (with a learning rate of 0.0002 and a beta_1 momentum value of 0.5) and the LeakyReLU [57] activation function were chosen because they are commonly used in the area of deep learning [58]. This simple architecture has 21,401 parameters for the discriminator and 31,305 for the generator, as one can see in the model summary in Table 1.

**Table 1:** Model summary for the vanilla GAN initial model.

Model: sequential_1		
Layer (type)	Output Shape	Param #
flatten_1 (Flatten)	(None, 5)	0
dense_1 (Dense)	(None, 200)	1200
leaky_re_lu_1 (LeakyReLU)	(None, 200)	0
dense_2 (Dense)	(None, 100)	20100
leaky_re_lu_2 (LeakyReLU)	(None, 100)	0
dense_3 (Dense)	(None, 1)	101
Total params: 21,401	Trainable params: 21,401	Non-trainable params: 0
Model: sequential_2		
Layer (type)	Output Shape	Param #
dense_4 (Dense)	(None, 100)	10100
leaky_re_lu_3 (LeakyReLU)	(None, 100)	0
dense_5 (Dense)	(None, 200)	20200
leaky_re_lu_4 (LeakyReLU)	(None, 200)	0
dense_6 (Dense)	(None, 5)	1005
reshape_1 (Reshape)	(None, 5, 1, 1)	0
Total params: 31,305	Trainable params: 31,305	Non-trainable params: 0

**Fig 4:**
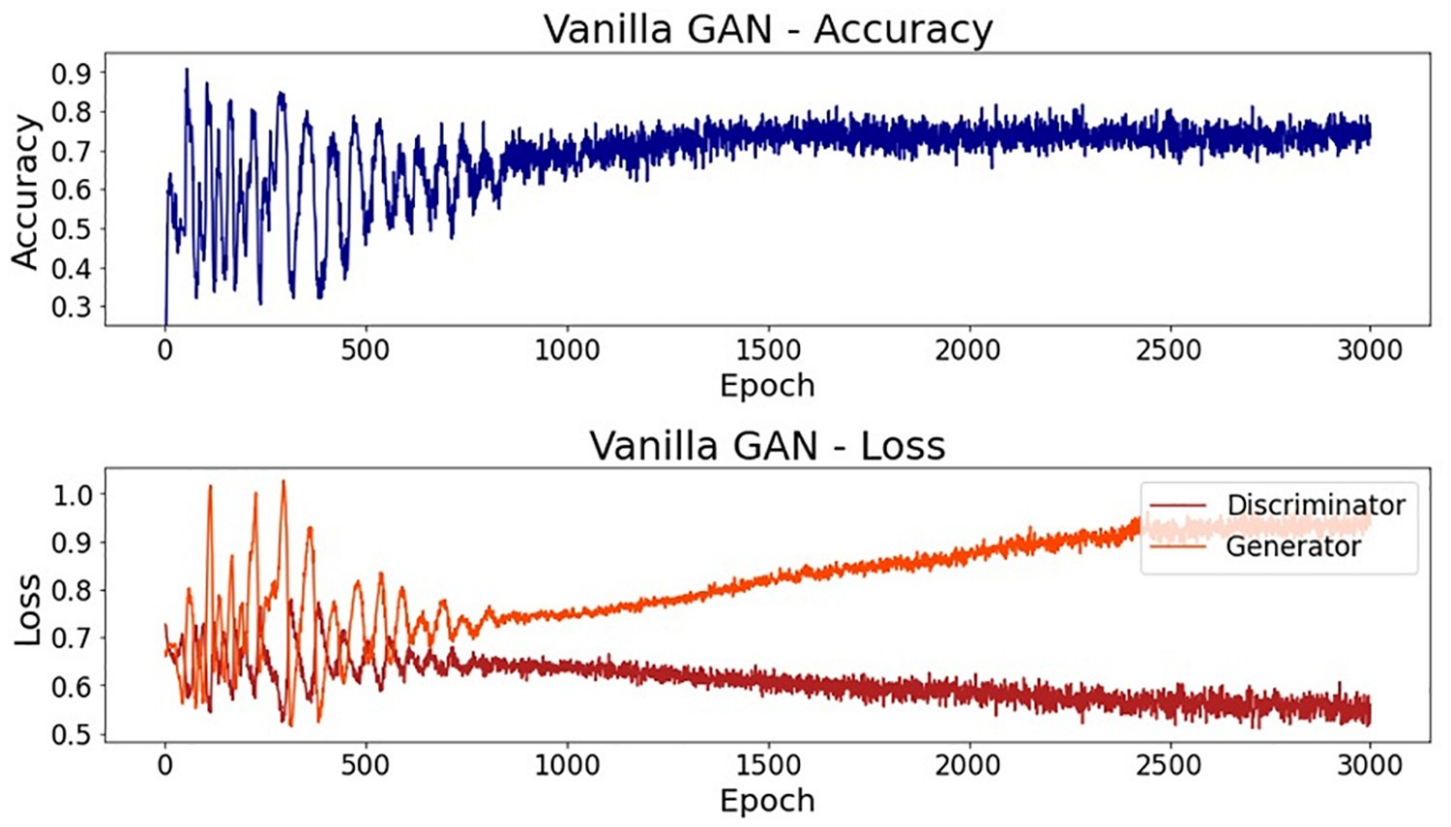
Vanilla GAN initial model: Accuracy and loss.

When analyzing the accuracy of this simpler model, the final value on epoch 3000 was 73.2%, a satisfactory result for this simple model. However, it is not necessarily expected that a good value for the discriminator accuracy will result in a good output of samples. In fact, for this first trial, the generated dataset was considerably different from the real one used in the training process. In the first subplot of Fig 4, it is possible to see a line plot for the accuracy along all of the epochs. It is also noteworthy that the accuracy presents high oscillations until epoch 900, approximately. After epoch 1000, the value stabilizes near 0.75. In the bottom subplot, it is possible to see the error for both the generator and the discriminator networks along the epochs. Here, even though the oscillation also decreases after epoch 900, approximately, it is not possible to see stable behavior.

**Fig 5:**
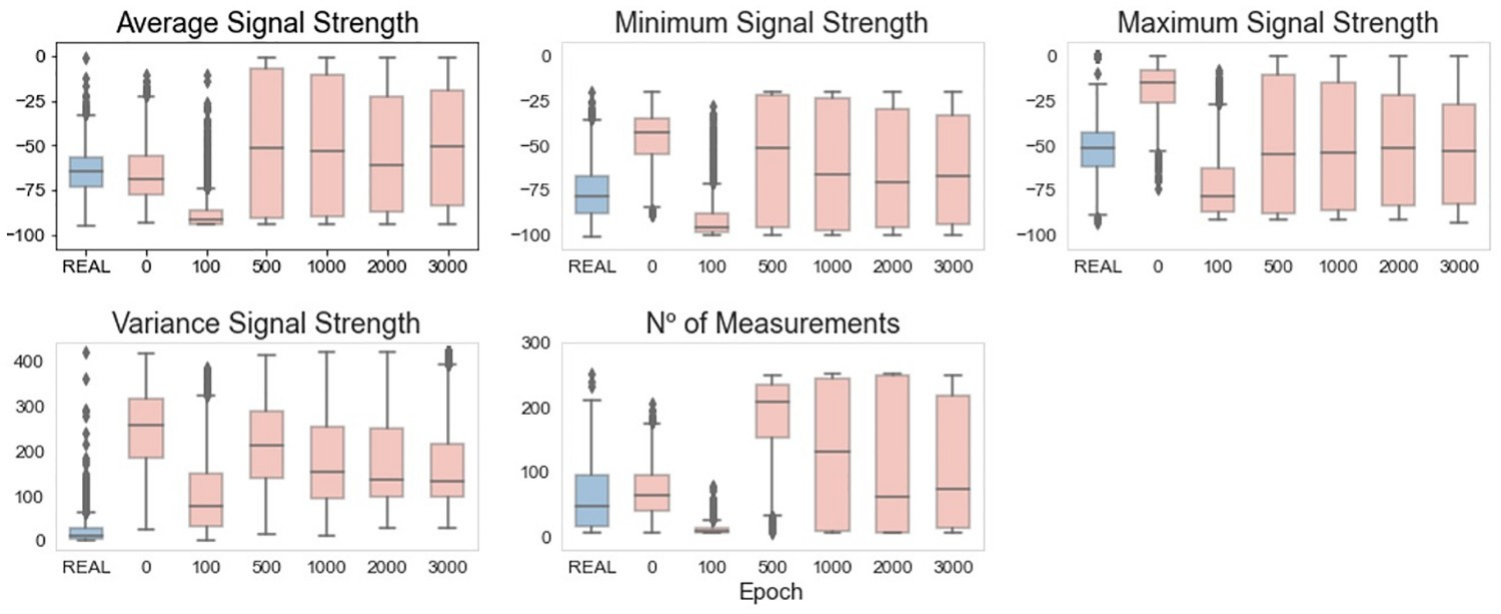
Vanilla GAN initial model: Boxplots of real and generated data.

To evaluate the quality of the generated dataset more practically, we performed a comparison between the distribution of each generated feature and the original distribution of the features. To make it easier to analyze the results along the training process, Fig 5 reports the boxplots of the different features in some selected epochs of the training process.

**Fig 6:**
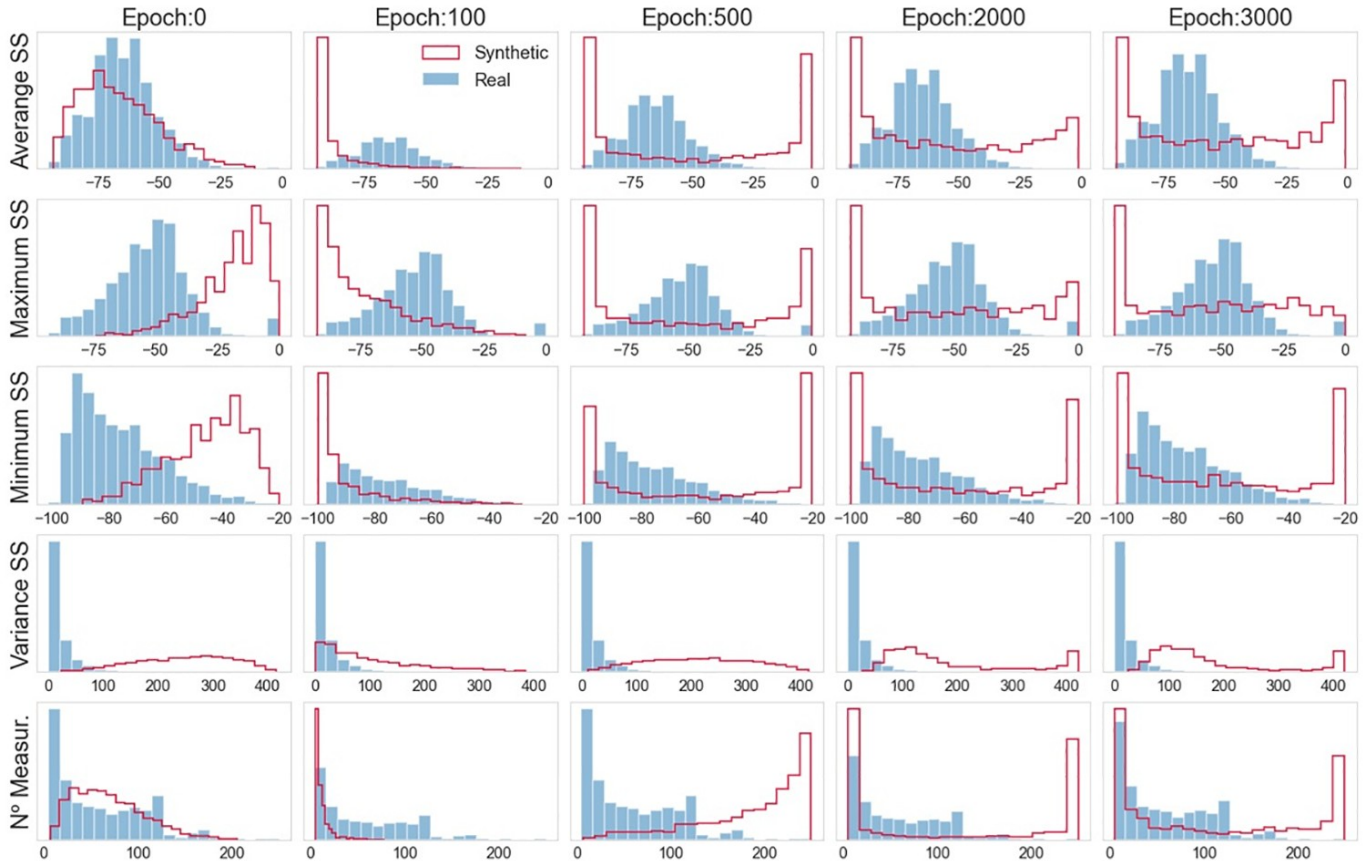
Vanilla GAN initial model: Histograms of real and generated data.

As it is possible to notice, at the end of the training process, the median value is close to the target median value (i.e., the one of the real distribution) for almost all of the features. However, for all of the features, the values’ dispersion is significantly greater for the generated data. A possible reason for that is the existence of extreme values in the distribution of the real features. The presence of these extreme values, as well as the asymmetric distribution that was not captured by the algorithm, is displayed in Fig 6. In particular, Fig 6 compares the distributions of the real and synthetic datasets through histograms drawn for some specific epochs.

To quantitatively measure whether the distribution of the synthetic data is a good approximation of the real distribution, we relied on the Kullback-Leibler (KL) divergence [59]. Intuitively, the KL divergence provides a measure of the amount of information we lose when we choose an approximation instead of the real distribution. By calculating the KL divergence for the distributions displayed in Fig 6 (at epoch 3000), we obtained the following values: *KL*(*REAL* ∥ *GAN*(*InitialModel*)): {’Average SS’: 0.887, ‘Minimum SS’: 0.717, ‘Maximum SS’: 0.582, ‘Variance SS’: 2.523, ‘N. Measurements’: 0.568}. Taking into account that the ideal KL divergence value is 0 (i.e., no information loss in replacing the real distribution with the synthetic one), the obtained values corroborated the qualitative analysis, thus suggesting that the considered GAN-based model cannot capture the complexity of the real data distribution.

**Fig 7:**
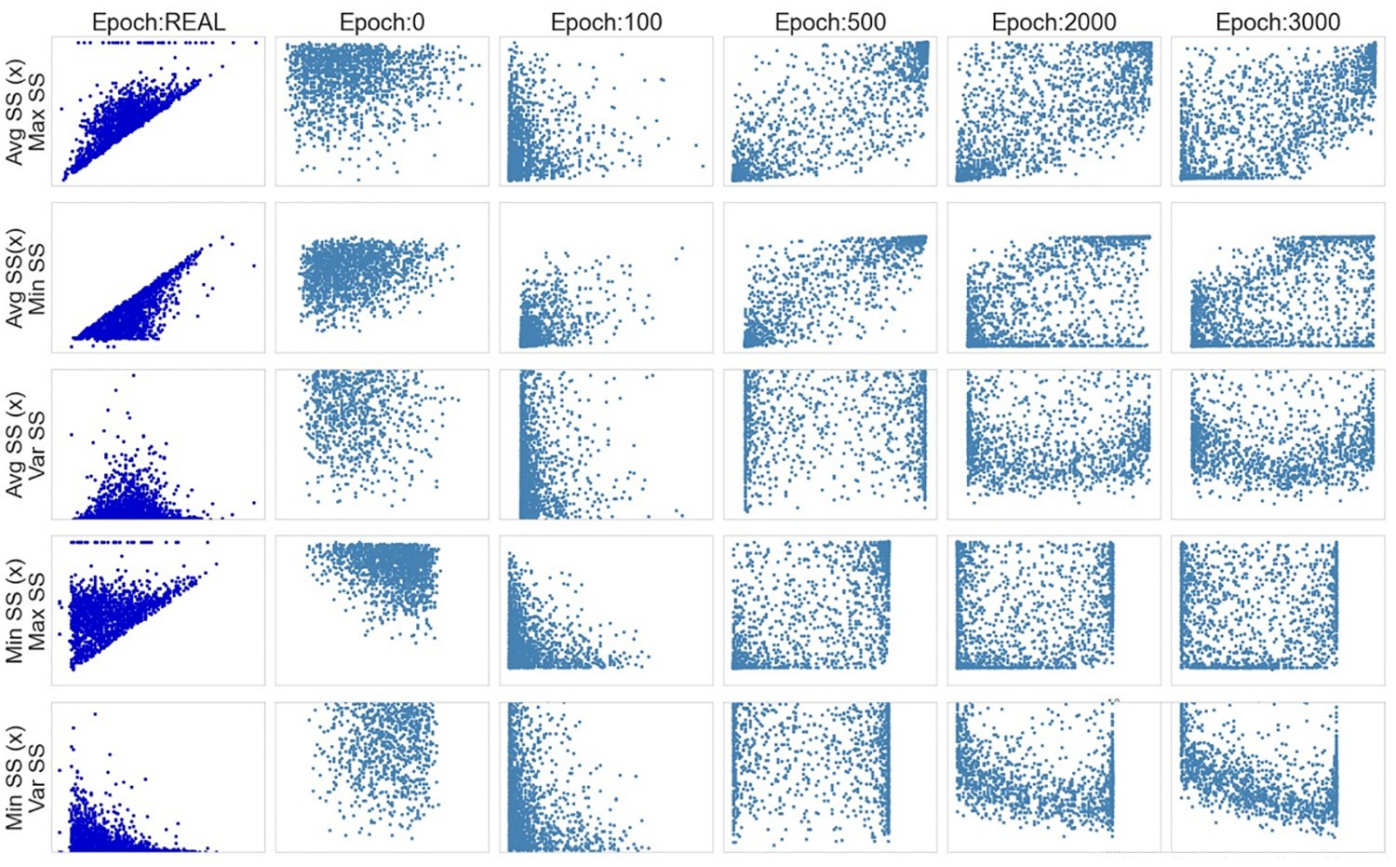
Vanilla GAN initial model: Scatter plots.

Another aspect to be considered when analyzing the generated data is the correlation between the features. To perform this analysis, scatter plots were drawn to compare the evolution of the variables’ correlation along the epochs. The scatter plots in Fig 7 show some of these associations, for the cases where an obvious type of relationship was expected, such as Minimum SS x Maximum SS, or where a more interesting association was identified. The axis values are the same for all of the plots along the epochs, always following the limits of the real data.

In the scatter plots it is possible to see that the generated datasets did not show any tendency or any kind of association between the variables. Instead, they kept showing a completely random distribution during the whole training process.

After this analysis, we decided to train the networks considering different values for the hyperparameters and analyze the new results. In the hyperparameter tuning phase, several options were tested including the following: increase the number of hidden layers, for the discriminator and for the generator; increase the number of neurons in each layer; use various activation functions; use other optimizers, considering different arguments (e.g., rate and/or momentum); use batch normalization, considering various values for the momentum argument; train the discriminator more times than the generator; consider different distributions and dimensions for the noise vector *z*; consider different batch sizes during the training process; consider min-max normalization; and train the discriminator model on real and synthetic data separately.

**Fig 8:**
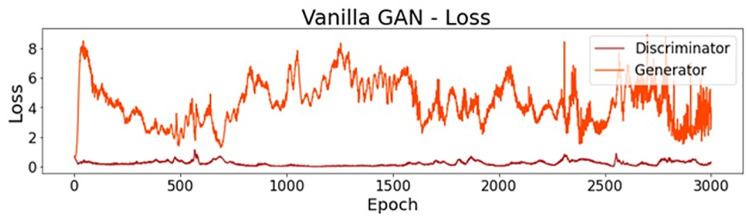
Vanilla GAN: Accuracy and loss for a model with mode collapse.

**Fig 9:**
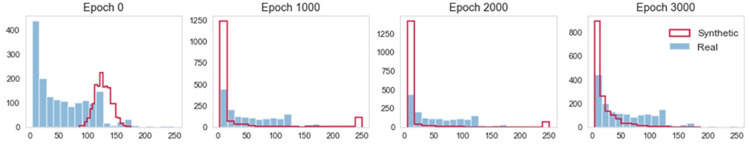
Vanilla GAN: Example of histograms for a model with mode collapse.

As expected, during the tuning process, some of the hyperparameters required more attention than others and, consequently, created the necessity of training the networks more times, considering different values. Different optimizers or different optimizers’ arguments, for example, showed a significant variation in network performance and outputted data and, for this reason, had to be tested many times. On the other hand, training the discriminator model with real and synthetic data separately presented better results compared to the other trial, independently of the other hyperparameters’ values. For some combinations of hyperparameters, the model presented the classic drawbacks of GANs, such as mode collapse or convergence failure. Fig 8, for example, shows the line plots of accuracy and loss for a model with model collapse. In Fig 9 it is possible to see that—for the feature “Nº of measurements”—almost all the of generated instances on epoch 3000 are concentrated between 0 and 50.

The final model that outputted a dataset similar to the real one was the model with the following structure and hyperparameter values:

Generator Model:

- Distribution of the noise vector *z*: Uniform(-1, 1)
- Dimension of the noise vector *z*: 500
- Number of layers: 4—the input layer, two hidden layers, and the output layer
- Number of neurons in each layer: input layer: 300; hidden layer 1: 600; hidden layer 2: 1200; output layer: 5
- Activation functions: input and hidden layers: LeakyReLU (alpha = 0.15);
- Output layer: Hyperbolic tangent
- Use of batch normalization: Yes, with a momentum = 0.8.

Discriminator Model:

- Number of layers: 4—the input layer, two hidden layers, and the output layer
- Number of neurons in each layer: input layer: 1200; hidden layer 1: 600; hidden layer 2: 300; output layer: 1
- Activation functions: input and hidden layers: LeakyReLU(alpha = 0.15);
- output layer: sigmoid
- Use of batch normalization: not used

Composite model and train process:

- Optimizer: Adam(0.0002, 0.3)
- Batch size: 220
- Number of epochs (stopping condition): 3000
- Number of updates of the discriminator per generator update: 1

The final model has 908,401 parameters for the first network and 1,066,505 for the second network, as one can see in the model summary in Table 2.

**Table 2:** Model summary for the vanilla GAN model.

Model: sequential_1		
Layer (type)	Output Shape	Param #
flatten_1 (Flatten)	(None, 5)	0
dense_1 (Dense)	(None, 1200)	7200
leaky_re_lu_1 (LeakyReLU)	(None, 1200)	0
dense_2 (Dense)	(None, 600)	720600
leaky_re_lu_2 (LeakyReLU)	(None, 600)	0
dense_3 (Dense)	(None, 300)	180300
leaky_re_lu_3 (LeakyReLU)	(None, 300)	0
dense_4 (Dense)	(None, 1)	301
Total params: 908,401	Trainable params: 908,401	Non-trainable params: 0
Model: sequential_2		
Layer (type)	Output Shape	Param #
dense_5 (Dense)	(None, 300)	150300
leaky_re_lu_4 (LeakyReLU)	(None, 300)	0
batch_normalization_1	(None, 300)	1200
dense_6 (Dense)	(None, 600)	180600
leaky_re_lu_5 (LeakyReLU)	(None, 600)	0
batch_normalization_2	(None, 600)	2400
dense_7 (Dense)	(None, 1200)	721200
leaky_re_lu_6 (LeakyReLU)	(None, 1200)	0
batch_normalization_3	(None, 1200)	4800
dense_8 (Dense)	(None, 5)	6005
reshape_1 (Reshape)	(None, 5, 1, 1)	0
Total params: 1,066,505	Trainable params: 1,062,305	Non-trainable params: 4,200

**Fig 10:**
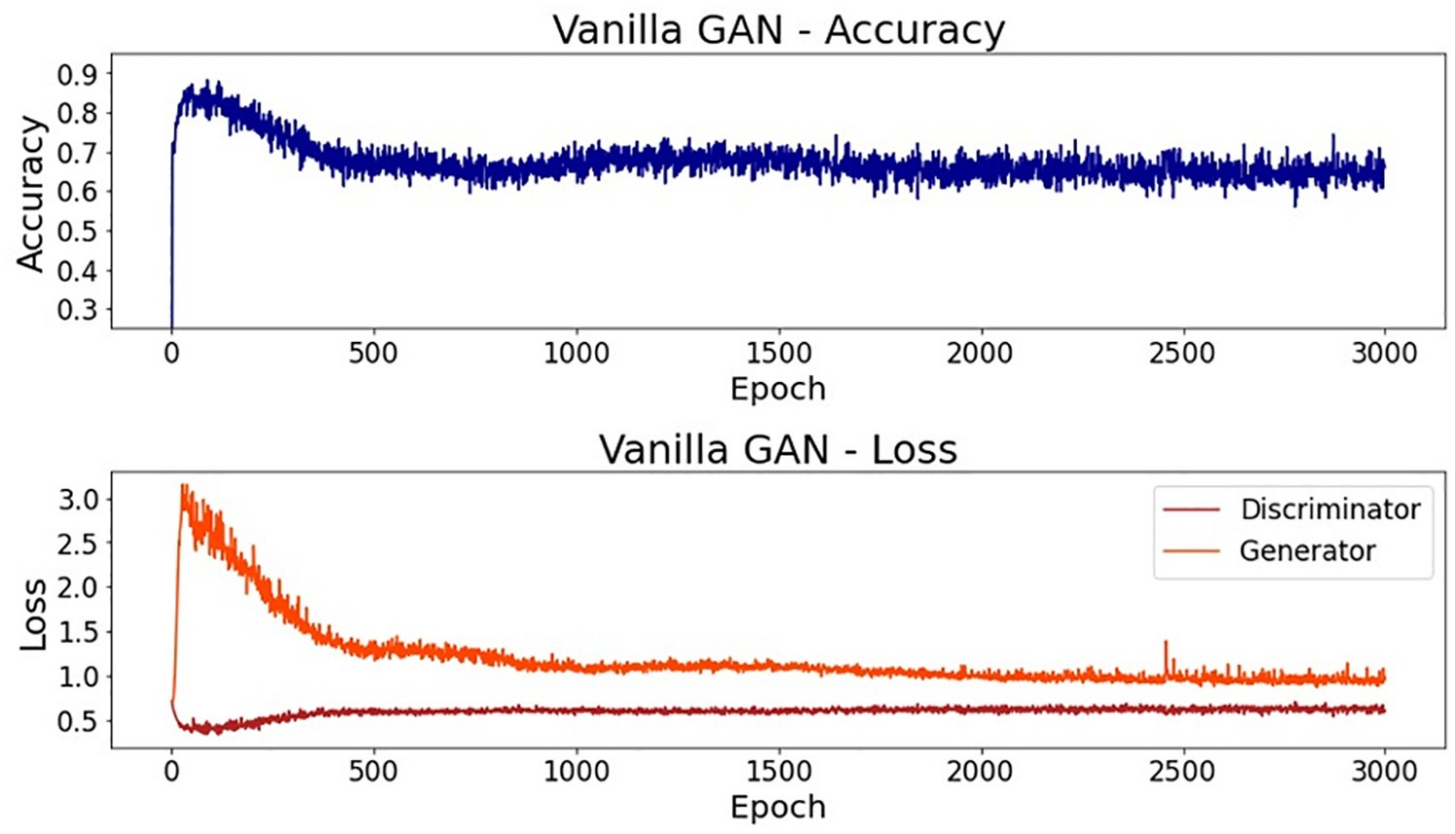
Vanilla GAN final model: Accuracy and loss.

Regarding feature scaling, better results were achieved using the min-max normalization. Fig 10 reports the accuracy along the epochs for this final model. As it is possible to notice, this accuracy decreases until epoch 500, approximately, and then gets stable near 0.65. The final accuracy, on epoch 3000, was 64.8%. This stability can also be observed for the loss lines.

**Fig 11:**
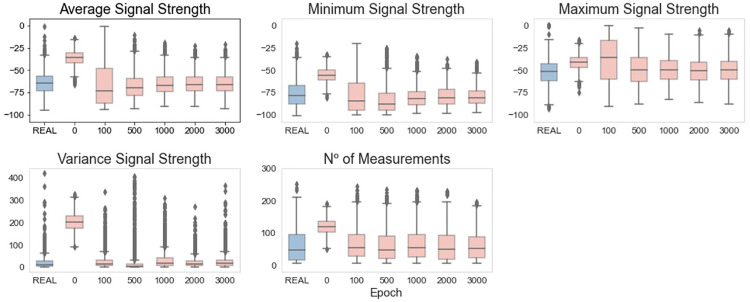
Vanilla GAN final model: Boxplots of real and generated data.

By analyzing the synthetic through the boxplots (Fig 11), it is possible to notice that, after epoch 1000, the values for the generated data are close to the values of the real data. The worst results are observed for the feature Minimum Signal Strength, which presents a smaller interquartile range. A common behavior observed in almost all the features is that the generated data do not have so many extreme values as the real data. In other words, the boxplots present fewer outliers.

**Fig 12:**
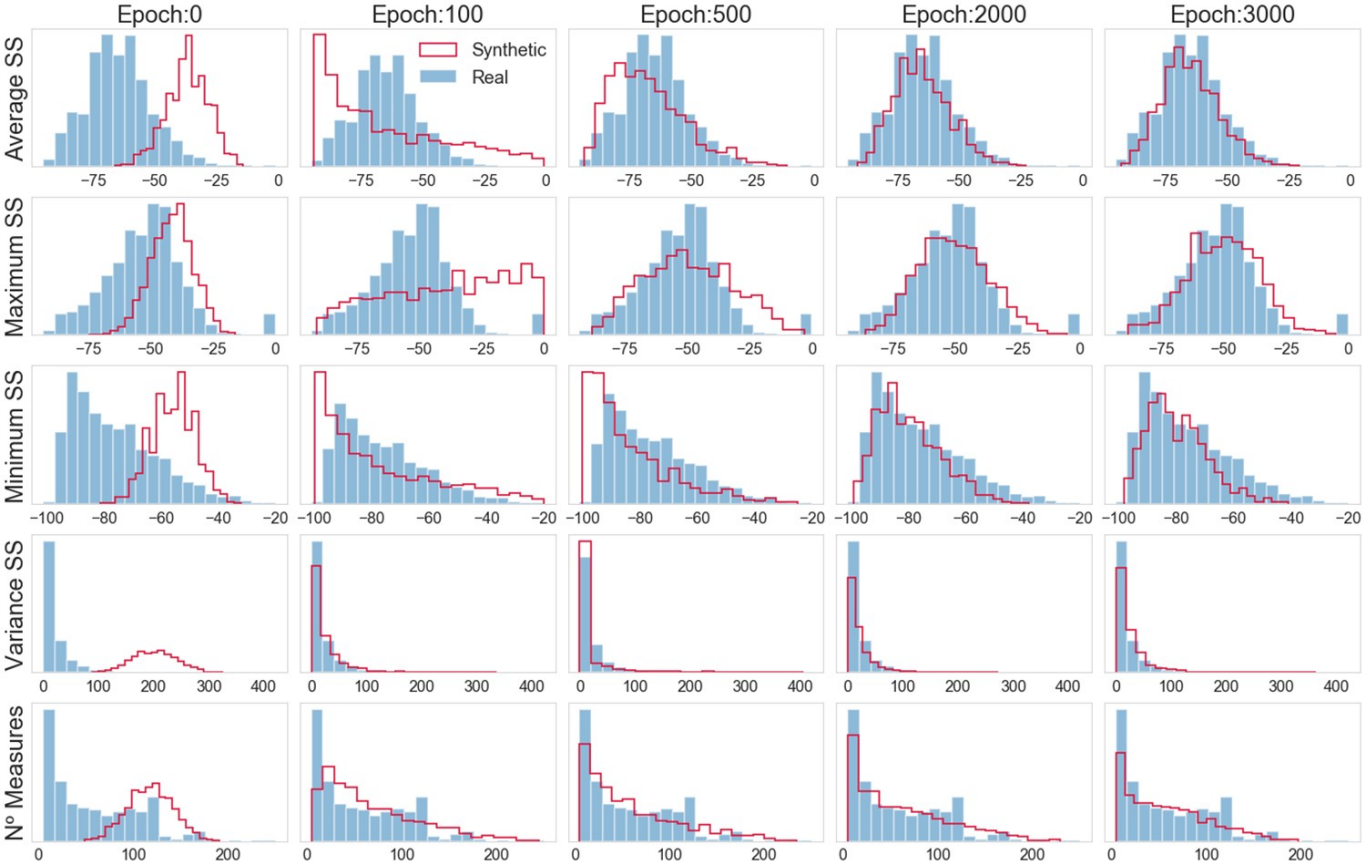
Vanilla GAN final model: Histograms of real and generated data.

When analyzing the data distribution through the histograms in Fig 12, good results are observed, especially after epoch 1000. In particular, for the considered features, the distribution of the synthetic data clearly overlaps the distribution of the real data. In other terms, the vanilla GAN can create synthetic data that mimic the distribution of the original data. Similar to what we did for the initial GAN-based model, to quantitatively measure whether the distribution of the synthetic data is a good approximation of the real distribution, we calculated the KL divergence for the distributions (obtained at epoch 3000) displayed in Fig 12. The KL values obtained are the following: *KL*(*REAL* ∥ *GAN*(*FinalModel*)): {’Average SS’: 0.041, ‘Minimum SS’: 0.041, ‘Maximum SS’: 0.08 ‘Variance SS’: 0.081, ‘N. Measurements’: 0.084}. As one can see, these KL values are significantly smaller than the ones achieved with the initial GAN-based model, thus strengthening the previous qualitative analysis. In particular, there is clear evidence of the suitability of the GAN model in providing an excellent approximation of the real distribution of Wi-Fi features.

**Fig 13:**
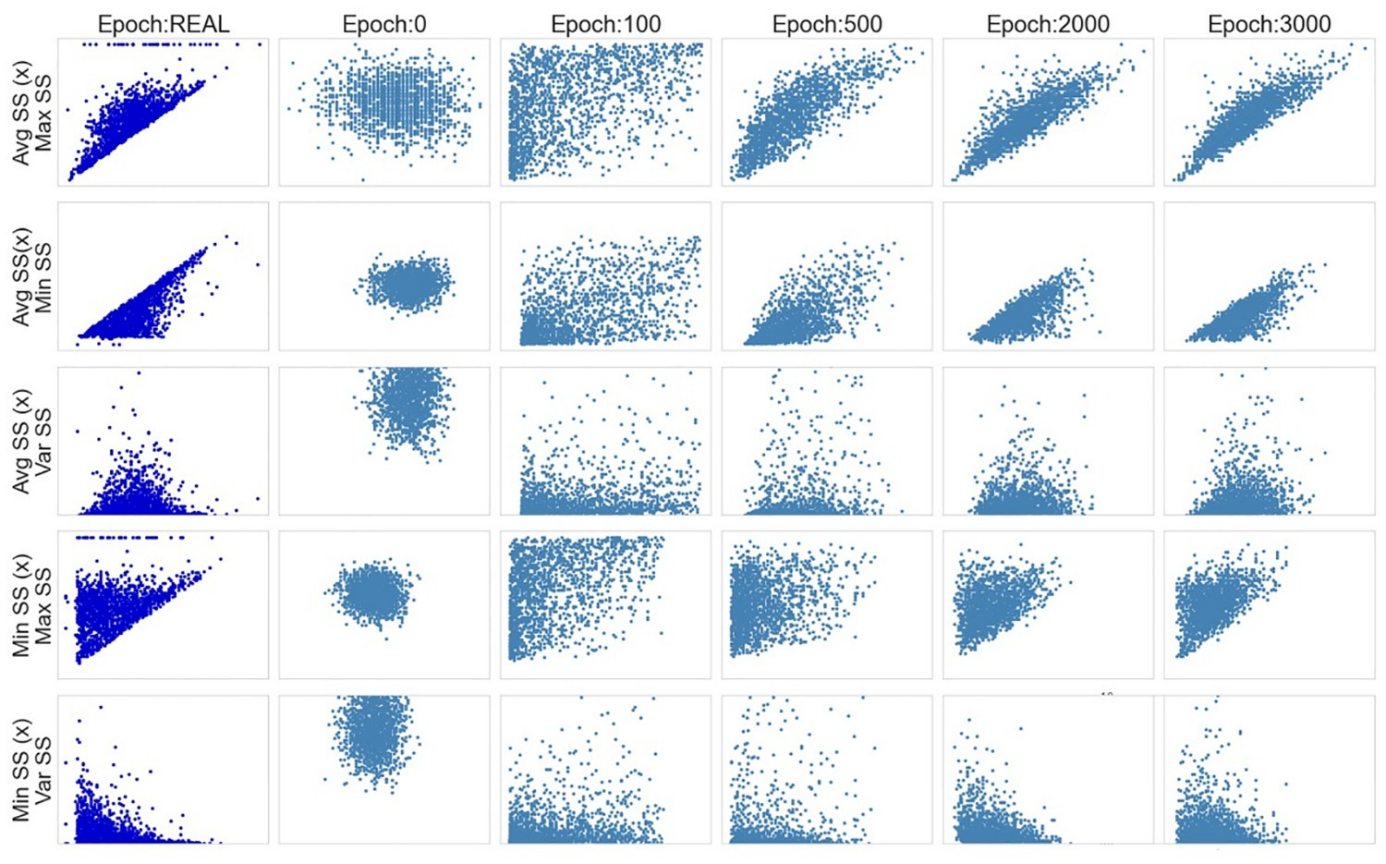
Vanilla GAN final model: Scatter plots.

In Fig 13, the scatter plots show the association between the generated features. As it is possible to see, the model can generally reproduce the same association between the synthetic features observed in the real ones. However, for a small number of cases, the generated instances did not follow the expected rules. For example, there are instances where the value of Min SS is greater than the value of Max SS. However, this phenomenon happens for a small number of observations.

Another issue that deserves attention is that the model was not able to mimic the little volume of values concentrated on zero for the variable Maximum SS, as it is possible to see in the histograms and in the scatterplots. However, these extreme values are not expected in the real world and their presence should be investigated in more detail. While the performance of the model is considered satisfactory from the perspective of the company, more complex topologies may improve these results. However, the choice of the final model must consider a trade-off between performance and computational effort (i.e., the time needed to train the model). This is particularly important considering that the model used in the production system of the company must be trained on a vast amount of data. Additionally, fine-tuning of the model may be needed from time to time if changes in the Wi-Fi regulation are introduced.

### 5.2 WGAN

As explained in the previous sections, the WGAN changes only the loss function of the networks in a GAN architecture. The cross-entropy loss is replaced with the EM distance, a score representing the “realness” or “fakeness” of an instance. It is not a number between 0 and 1 that can be interpreted as a probability, as in GANs. As it is the only difference between the architectures, the same logic of analysis was followed to evaluate the WGAN results. Thus, the training process started considering a simple network. After realizing that it did not present good results, which was expected considering the previous analysis of the vanilla GAN, we modified the WGAN by considering a structure similar to the one used for the final model of the vanilla GAN architecture. During the tuning of the hyperparameters, special attention was given to some specific recommendations pointed out in the original paper. The authors recommended, for example, the use of the RMSprop optimizer with a small learning rate. They also recommend updating the critic network more times than the generator. Hyperparameter tuning requires patience in all neural networks and the same was true for WGANs. Even though one of the biggest advantages of the WGAN is that it can provide a loss function that correlates with the quality of the generated data and facilitates hyperparameter optimization, it was necessary to spend a significant amount of time on this step. In some scenarios, mode collapse and convergence failure happened. After the hyperparameter tuning process, the final model was the following: Generator Model:

- Distribution of noise vector *z*: Uniform(-1, 1)
- Dimension of noise vector *z*: 100
- Number of layers: 4—the input layer, two hidden layers, and the output layer
- Number of neurons in each layer: input layer: 250; hidden layer 1: 500; hidden layer 2: 1000; output layer: 5
- Activation functions: input and hidden layers: LeakyReLU(alpha = 0.2); output layer: hyperbolic tangent
- Use of batch normalization: Yes, with a momentum = 0.8.

Critic Model:

- Number of layers: 4—the input layer, two hidden layers, and the output layer
- Number of neurons in each layer: input layer: 1000; hidden layer 1: 500; hidden layer 2: 250; output layer: 1
- Activation functions: input and hidden layers: LeakyReLU(alpha = 0.2); output layer: linear
- Use of batch normalization: not used
- Weight clipping: 0.05

Composite model and train process:

- Optimizer: RMSprop(lr = 0.00007)
- Batch size: 220
- Number of epochs (stopping condition): 3000
- Number of critic updates per generator update: 2

Also in this case, better results were observed using the min-max normalization feature scaling. Even though the chosen model updates the critic network two times more than it does the generator, the model with the same number of updates also presented satisfactory results. This is a hyperparameter that can be adjusted to reduce the training time if necessary. As it is possible to see from the summary in Table 3, the resulting model structure has 632,001 parameters for the first network, the critic, and 663,755 for the second, the generator.

**Table 3:** Model summary for the WGAN model.

Model: sequential_1		
Layer (type)	Output Shape	Param #
flatten_9 (Flatten)	(None, 5)	0
dense_65 (Dense)	(None, 1000)	6000
leaky_re_lu_49 (LeakyReLU)	(None, 1000)	0
dense_66 (Dense)	(None, 500)	500500
leaky_re_lu_50 (LeakyReLU)	(None, 500)	0
dense_67 (Dense)	(None, 250)	125250
leaky_re_lu_51 (LeakyReLU)	(None, 250)	0
dense_68 (Dense)	(None, 1)	251
Total params: 632,001	Trainable params: 632,001	Non-trainable params: 0
Model: sequential_2		
Layer (type)	Output Shape	Param #
dense_69 (Dense)	(None, 250)	25250
leaky_re_lu_52 (LeakyReLU)	(None, 250)	0
batch_normalization_25	(None, 250)	1000
dense_70 (Dense)	(None, 500)	125500
leaky_re_lu_53 (LeakyReLU)	(None, 500)	0
batch_normalization_26	(None, 500)	2000
dense_71 (Dense)	(None, 1000)	501000
leaky_re_lu_54 (LeakyReLU)	(None, 1000)	0
batch_normalization_27	(None, 1000)	4000
dense_72 (Dense)	(None, 5)	5005
reshape_9 (Reshape)	(None, 5, 1, 1)	0
Total params: 663,755	Trainable params: 660,255	Non-trainable params: 3,500

**Fig 14:**
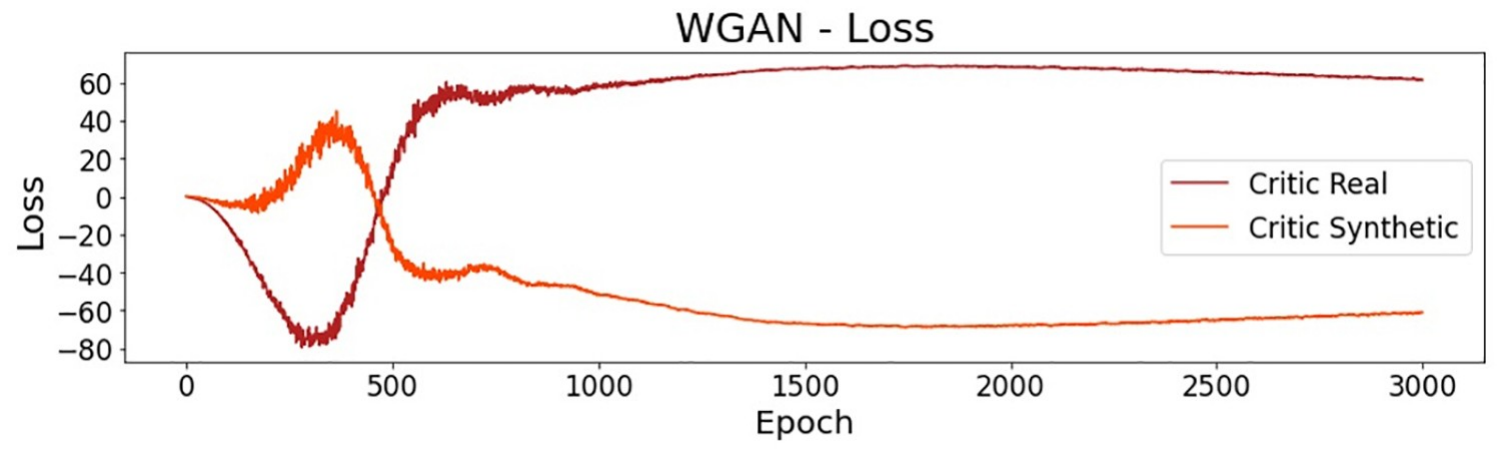
WGAN: Critic and generator loss.

Fig 14 presents the loss for the critic network on real and synthetic samples. Both of the curves become stable from epoch 1500 (approximately) until the end of the training process. As the critic loss for generated data (Critic Synthetic) decreases, more realistic synthetic instances are expected.

**Fig 15:**
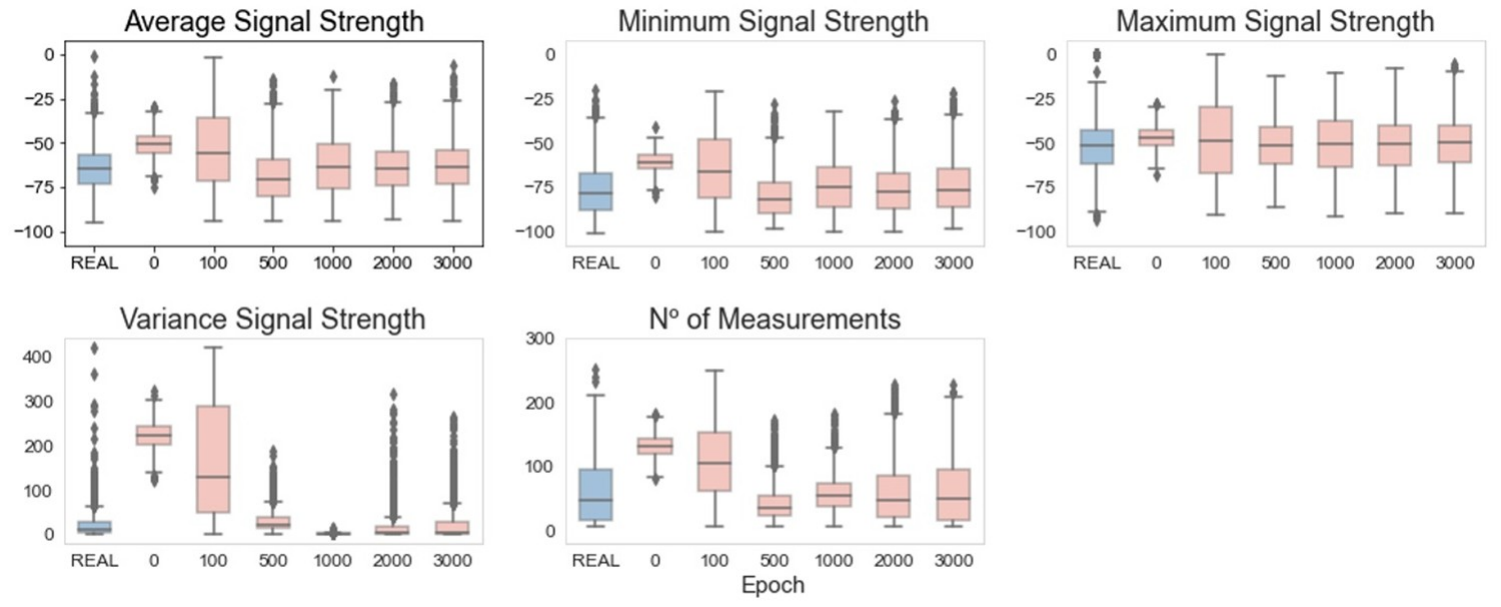
WGAN: Boxplots of real and generated data.

Using the boxplots in Fig 15 to analyze the synthetic data, it is possible to see that, after epoch 2000 and for all of the considered features, the generated data present a behavior very similar to the real data. Although this behavior appeared earlier (at epoch 1000) in the vanilla GAN model, the WGAN seems to be more suitable in the simulation of extreme values.

**Fig 16:**
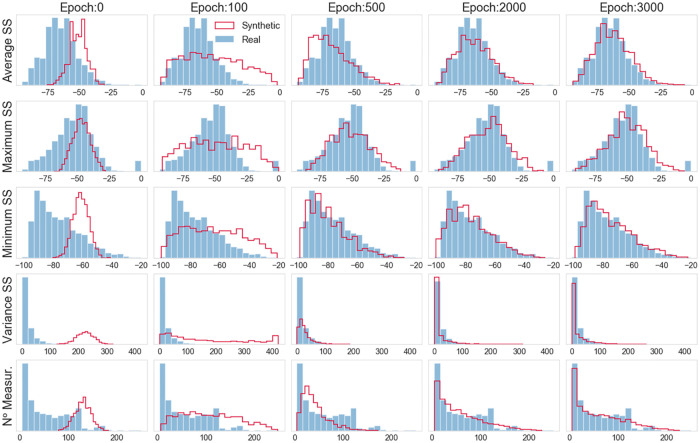
WGAN: Histograms of real and generated data.

The the histograms in Fig 16 further corroborate the analysis. From these plots, it is also possible to see that the synthetic data distributions overlap the real distributions, and the WGAN can provide a better distribution (with respect to the vanilla GAN) of the most asymmetric features. Concerning the quantitative analysis, we calculated the KL for the distributions (obtained at epoch 3000) displayed in Fig 16. The KL values obtained are the following: *KL*(*REAL* ∥ *GAN*(*FinalModel*)): {’Average SS’: 0.043, ‘Minimum SS’: 0.042, ‘Maximum SS’: 0.93, ‘Variance SS’: 0.077, ‘N. Measurements’: 0.095}. Also, in this case, clear evidenceexists of the suitability of the WGAN model for providing an excellent approximation of the real distribution of Wi-Fi features. Moreover, the KL divergence values achieved with the GAN and WGAN models are comparable. Thus, the two GANs perform similarly on the task at hand.

**Fig 17:**
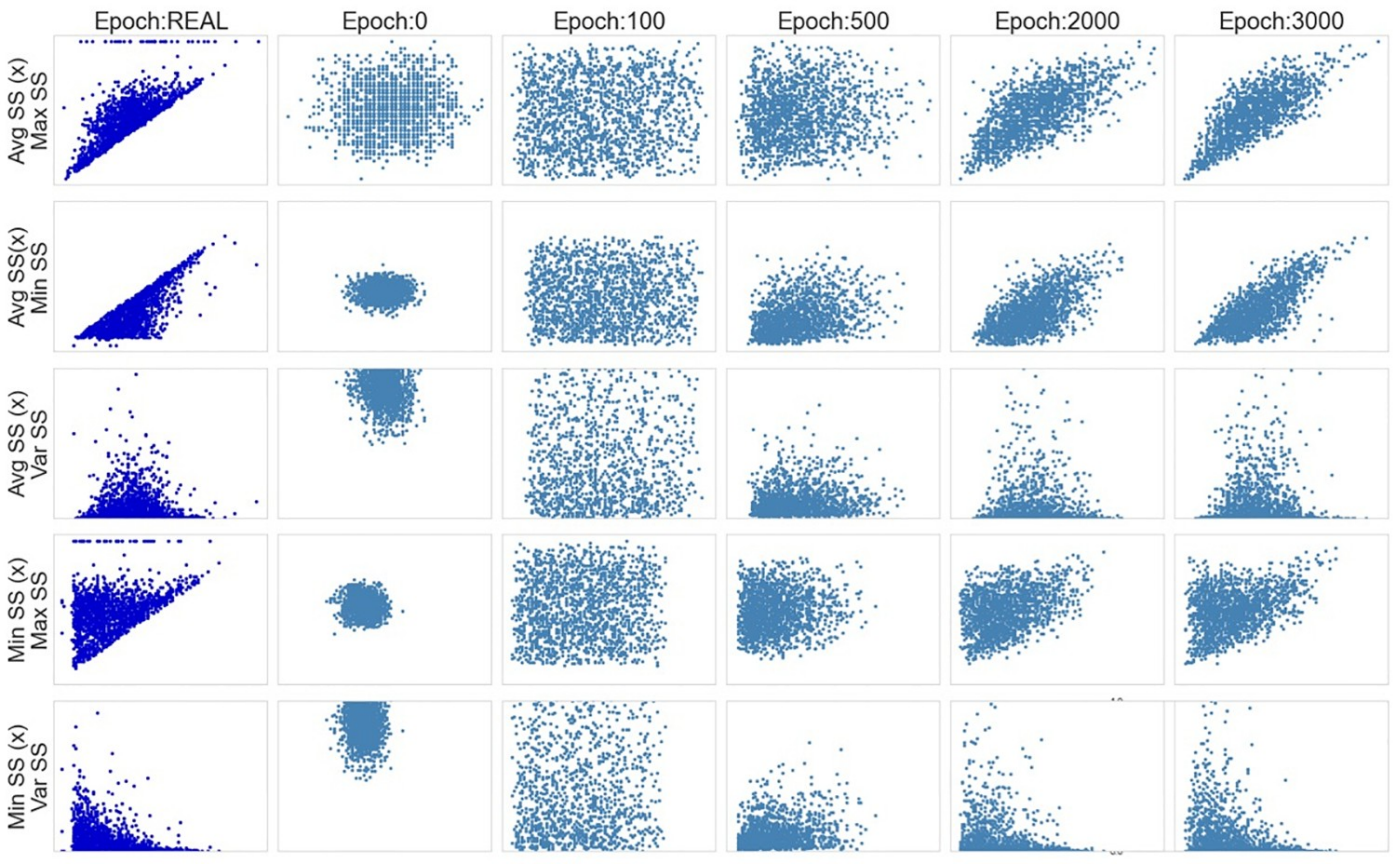
WGAN: Scatter plots.

Finally, the scatterplots in Fig 17, show the model’ suitability for reproducing the same associations, observed in the real data, between the synthetic features. Still, was the case for the vanilla GAN model, in a small number of cases, the value of Min SS is higher than the value of Avg SS or Max SS.

As in the vanilla GAN architecture, the WGAN model cannot mimic the observations with a value of zero for the variable Maximum SS (as it is possible to see on the scatter plots in lines 1 and 4 of Fig 17). However, as previously discussed, these extreme values should be investigated in more detail because they are not expected from working devices.

### 5.3 Analysis of the GANs’ topology

Section 5.1 and Section 5.2 present the results that the considered GAN-based models achieved after the optimization of different hyperparameters. Interestingly, in both cases, an architecture characterized by two hidden layers was deemed sufficient to achieve robust results. To empirically demonstrate how the number of hidden layers and the number of neurons affect the performance of the GAN-based models for the problem at hand, we performed a set of experiments. In particular, for both the vanilla GAN and the WGAN models, we executed the following tests: 1) increase the number of hidden layers; 2) increase the number of neurons, and 3) simultaneously increase the number of hidden layers and neurons. This analysis is aimed at corroborating the choice made in the previous sections. In particular, we show that increasing the value of the aforementioned hyperparameters does not produce any performance advantage with respect to the models discussed in Section 5.1 and Section 5.2.

We first present the results of this analysis for the vanilla GAN. Table 4 summarizes the topology of the vanilla GAN architectures considered in this test.

**Table 4:** Topology of the vanilla GANs considered in the analysis of the hyperparameters (number of layers and number of neurons). For each topology, the table specifies the main change with respect to the GAN considered in Section 5.1, as well as the number of neurons in each layer. G stands for generator, and D for discriminator. The hidden layers are indicated as H1, H2, and H3. The input layer is denoted as In and the output layer as Out.

ID	Change	Vanilla GAN—Topology
1	More layers	G: In: 200—H1: 400—H2: 800—H3: 1600—Out: 5
		D: In: 1600—H1: 800—H2: 400—H3: 200—Out: 1
2	More neurons	G: In: 600—H1: 1200—H2: 2400—Out: 5
		D: In: 2400—H1: 1200—H2: 600—Out: 1
3	More layers and neurons	G: In: 350—H1: 700—H2: 1000—H3: 2000—Out: 5
		D: In: 2000—H1: 1000—H2: 700—H3: 350—Out: 1

To assess the results that each configuration achieved and compare them against the performance that the model of Section 5.1 achieved, we rely on the analysis of the distribution and the KL divergence. In this way, we have both a qualitative and a quantitative evaluation. Concerning the first topology, S1 Fig displays the histograms of real and generated data. As one can see, the histograms look similar to the ones displayed in Fig 12. Thus, adding one layer to the topology discussed in Section 5.1 does not provide any significant advantage. To corroborate this visual comparison, we calculate the KL divergence value for the different features, as we did in the previous sections. The KL values obtained are the following: *KL*(*REAL* ∥ *GAN*(*ID*1)): {’Average SS’: 0.068, ‘Minimum SS’: 0.294, ‘Maximum SS’: 0.195 ‘Variance SS’: 0.163, ‘N. Measurements’: 0.044}. By comparing these values with the one obtained with the selected vanilla GAN model, we can state that the performance of the model does not improve: despite the feature ‘N. Measurements’, the KL divergence values obtained for the distributions in S1 Fig are worse than the ones calculated for the distribution reported in Fig 12. This analysis can be extended to the vanilla GAN topology obtained by adding more neurons (topology Id 2 of Table 4). S2 Fig displays the histograms of real and generated data. Also, in this case, the visual analysis does not allow one to determine any competitive advantage of the topology with added neurons compared with that of Section 5.1. This observation is confirmed by the KL divergence values, which are the following: *KL*(*REAL* ∥ *GAN*(*ID*2)): {’Average SS’: 0.099, ‘Minimum SS’: 0.229, ‘Maximum SS’: 0.078 ‘Variance SS’: 0.146, ‘N. Measurements’: 0.275}. All of the values (despite the ‘Maximum SS’) are worse than the ones achieved with the final vanilla GAN model presented in Section 5.1. Taking into account the analysis of topologies ID 1 and ID 2 described in Table 4, we can expect that adding, at the same time, more neurons and more layers will not result in better performance. As displayed in S3 Fig, for topology ID 3, the histograms of the real and generated data are similar to those of Fig 12. For this vanilla GAN, we obtained the following KL divergence values: *KL*(*REAL* ∥ *GAN*(*ID*3)): {’Average SS’: 0.215, ‘Minimum SS’: 0.039, ‘Maximum SS’: 0.14 ‘Variance SS’: 0.168, ‘N. Measurements’: 0.296}. Thus, in this case, the quantitative analysis also suggests that adding more layers and neurons to the selected vanilla GAN model offers no advantage. All in all, the qualitative analysis suggests that all of the considered vanilla GAN topologies perform similarly in terms of the distributions of real and generated data. Nonetheless, the quantitative analysis performed with the KL divergence values indicated that the best match between the distributions of the real and generated data is obtained with the final vanilla GAN model of Section 5.1.

The same analysis was performed for the WGAN-based model. Table 5 summarizes the topology of the WGAN architectures considered in this test.

**Table 5:** Topology of the WGANs considered in the analysis of the hyperparameters (number of layers and number of neurons). For each topology, the table specifies the main change with respect to the GAN considered in Section 5.2, as well as the number of neurons in each layer. G stands for generator, and D stands for discriminator. The hidden layers are indicated as H1, H2, and H3. The input layer is denoted as In and the output layer as Out.

ID	Change	Vanilla GAN—Topology
1	More layers	G: In: 200—H1: 400—H2: 800—H3: 1600—Out: 5
		D: In: 1600—H1: 800—H2: 400—H3: 200—Out: 1
2	More neurons	G: In: 350—H1: 700—H2: 1400—Out: 5
		D: In: 1400—H1: 700—H2: 350—Out: 1
3	More layers and neurons	G: In: 300—H1: 600—H2: 1000—H3: 1500—Out: 5
		D: In: 1500—H1: 1000—H2: 600—H3: 300—Out: 1

Focusing on the first topology of Table 5, S4 Fig shows the histograms of real and generated data. As one can see, the histograms are comparable to the ones presented in Fig 16. Thus, as was the cae for the vanilla GAN architecture, adding one layer to the topology discussed in Section 5.2 does not provide any significant advantage in terms of the quality of the generated data. The KL values obtained are the following: *KL*(*REAL* ∥ *GAN*(*ID*1)): {’Average SS’: 0.067, ‘Minimum SS’: 0.052, ‘Maximum SS’: 0.051 ‘Variance SS’: 0.099, ‘N. Measurements’: 0.097}. By comparing these values with the ones obtained with the WGAN model of Section 5.2, we can state that the performance of the WGAN model does not improve with the addition of a hidden layer. Despite the feature ‘Maximum SS’, the KL divergence values obtained for the distributions in S4 Fig are worse than the ones calculated for the distribution reported in Fig 16. The second WGAN topology of Table 5 was obtained by adding more neurons to the WGAN of Section 5.2. S5 Fig reports the histograms of real and generated data. Also in this case, the qualitative analysis suggests that the performance of the WGAN model does not improve compared with the WGAN of Section 5.2. This result is confirmed by the values of the KL divergence. In particular, the KL divergence values extracted from the histograms of S5 Fig are the following: *KL*(*REAL* ∥ *GAN*(*ID*1)): {’Average SS’: 0.052, ‘Minimum SS’: 0.042, ‘Maximum SS’: 0.097 ‘Variance SS’: 0.084, ‘N. Measurements’: 0.067}. In particular, we can see that, although the values are comparable to the ones achieved from the analysis of Fig 16, the second topology of Table 5 produces a better KL value for the feature ‘N. Measurements’. All in all, for the first topology, both the quantitative and the qualitative analysis show a substantially comparable performance to that of the WGAN model of Section 5.2. Finally, the third topology (ID 3) was obtained by increasing both the number of hidden layers and neurons. S6 Fig displays the histograms of real and generated data. Similarly to the previously analyzed WGAN topologies, the histograms are comparable to the ones presented in Fig 16. The KL values obtained are the following: *KL*(*REAL* ∥ *GAN*(*ID*1)): {’Average SS’: 0.218, ‘Minimum SS’: 0.099, ‘Maximum SS’: 0.105 ‘Variance SS’: 0.12, ‘N. Measurements’: 0.14}. This topology presents the poorest KL divergence values among the considered WGAN topologies. Thus, it seems that adding more layers and neurons (i.e., considering more complex WGAN topologies) does not provide any competitive advantage for the problem at hand.

To summarize, this analysis strengthens the choice of the topologies of the vanilla GAN and WGAN models presented in Sections 5.1 and 5.2. In particular, by increasing the model’s complexity (i.e., number of neurons and hidden layers), the performance of both vanilla GAN and WGAN models does not improve.

### 5.4 Final model selection

**Fig 18:**
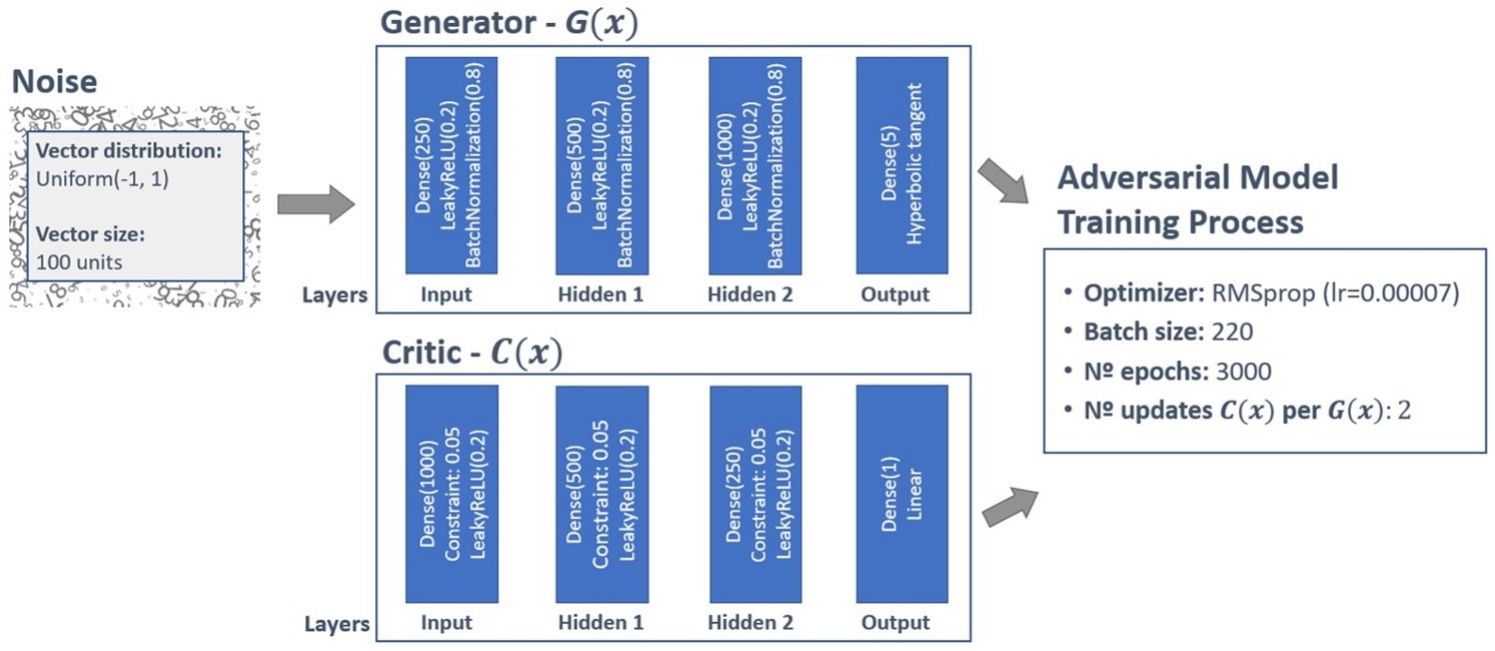
General view of the final model.

Training GAN is a process that requires experience and can easily fail. In this work, we considered several topologies (i.e., characterized by different hyperparameters) for the vanilla GAN and the WGAN, and we ended up with two models. Both the models can produce synthetic data that match the distribution of the original data. Although the WGAN is more suitable for modeling extreme data, it is difficult to state that the WGAN is the model that the company must select as a tool that will be made available to the partner companies responsible for testing the quality of the Wi-Fi signal. For this reason, in this section, we present the strategy we adopted for selecting the final model. The idea is to use a well-known classification algorithm for distinguishing between real and synthetic instances. Here, the chosen classifier is the random forest, a machine learning ensemble algorithm that consists of a collection of decision trees. This is a commonly used machine learning technique for addressing classification tasks, due to the good quality results it can achieve, as well as its robustness to overfitting. For this approach, new samples of synthetic data were generated considering both models (i.e., vanilla GAN and WGAN). For each model, we created a number of synthetic instances equal to the size of the real dataset. Thus, we ended up with two datasets: one containing the original data and the data created with the vanilla GAN, and the second containing the original data and the data created with the WGAN. Subsequently, we divided the two datasets into training (70%) and test sets (30%) and we trained two random forests, one trying to distinguish the real data from the synthetic instances generated via the GAN model, and the other trying to distinguish the real data from the data generated via the WGAN model. To ensure the robustness of the results, we trained 30 random forests for each of the two considered datasets. Moreover, to avoid any bias related to the choice of the synthetic dataset, we repeated the process considering 30 synthetic datasets. Thus, we trained a total of 900 random forest for each GAN-based model. For the random forest trained considering the data from the GAN model, the best models obtained an accuracy of 73.1%on the test data. The average accuracy (across the 30 synthetic datasets) was 75.1% with a standard deviation of 1%. For the random forest trained considering the data from the WGAN, the best model returned an accuracy of 72.1%. The average accuracy (across the 30 synthetic datasets) was 73.5% with a standard deviation of 0.8%. Even though these accuracies are higher than the ideal value, 50%, the descriptive analysis showed that the generated data present a behavior similar to the behavior of the real data. Thus, it is possible to conclude that both models present comparable results. The WGAN was chosen as the final model because it seemed slightly better in the descriptive analysis, at least in the interpretation of the domain experts. Fig 18 reports a schematic representation of the WGAN final model. It shows the model’s structure as well as the values of the hyperparameters.

## 6 Conclusions

Wireless networks represent a fundamental technology for ensuring reliable communications. With the rising popularity of mobile devices and the increasing amount of data these devices produced and shared, service providers must update the networks’ infrastructures to maximize the quality of the service that users receive. The networks’ complexity requires the use of advanced intelligent techniques to handle and optimize different tasks. Deep learning gained popularity in the field of wireless communication due to its ability to discover complex patterns by analyzing a vast amount of data, and it usually provided better performance compared with standard machine learning methods [3]. Despite the excellent results that deep learning models have achieved in this area, internet service providers are continuously looking for solutions to avoid service interruption and to solve possible connection troubles as soon as possible. Ensuring high-quality service is fundamental for keeping their customer portfolios. With this in mind, it is common to establish partnerships with specialized technology companies that deliver software services to monitor the networks and to identify faults and respective solutions. A common barrier that these specialized companies face is a lack of data to develop and test their products. This paper investigated the use of GANs for generating synthetic telecommunication data related to Wi-Fi signal quality. First, we developed and trained two GAN architectures, namely the vanilla GAN and the WGAN. Subsequently, for assessing the suitability of GANs for the task at hand, synthetic data were qualitatively and quantitatively compared with the real Wi-Fi networks’ data. Experimental results indicated that both models can generate synthetic data that match the real data distribution. In particular, the distribution of the synthetic data overlaps the distribution of the real data, for all of the considered features. Moreover, the considered generative models can reproduce the same associations, observed for the features, between the synthetic features. The WGAN was chosen as the final model, but both models are suitable for addressing the problem at hand. A second study corroborated the results of this analysis. In this study, a random forest-based classifier was used to discriminate between real and synthetic data: the poor classification accuracy indicated that the classifier cannot distinguish real data from synthetic data, thus strengthening the GAN architectures’ suitability for the generation of synthetic Wi-Fi network’s KPIs. Software companies can take advantage of this paper findings to build better automatic systems for monitoring the quality of Wi-Fi networks by using as much data as they need, thus enhancing their analyses’ reliability. On the other hand, as a result of this effort, the final user may experience a better quality of service and fewer service interruptions. This study paves the way for possible future works. In particular, when we developed the GAN-based model and reviewed the existing literature, we realized that the field of generative models evolved quickly in recent years. Despite this effort, almost all the new GAN architectures were proposed to address specific problems in the image generation field (like super-resolution) or to overcome the limitations of existing architectures concerning the generation of good-quality synthetic images. In other words, we believe it is fundamental to improve GANs’ ability to generate synthetic features in domains beyond image analysis. Based on the experience of this study, the need exits for a training process that can guarantee the convergence of the architecture by dynamically modifying the hyperparameters of the GAN or by considering more advanced loss functions. From a practical perspective, we aim to extend this study by considering more Wi-Fi-related features and more CPEs. With the comprehensive coverage of the domain of the original features, we may obtain a more robust GAN model.

## Supporting information

S1 Fig Vanilla GAN, model ID 1 (description in Table 4): Histograms of real and generated data. (TIF)

S2 Fig Vanilla GAN, model ID 2 (description in Table 4): Histograms of real and generated data. (TIF)

S3 Fig Vanilla GAN, model ID 3 (description in Table 4): Histograms of real and generated data. (TIF)

S4 Fig WGAN, model ID 1 (description in Table 5): Histograms of real and generated data. (TIF)

S5 Fig WGAN, model ID 2 (description in Table 5): Histograms of real and generated data. (TIF)

S6 Fig WGAN, model ID 3 (description in Table 5): Histograms of real and generated data. (TIF)

S1 Dataset Dataset used to train the GAN-based models considered in this study. The dataset contains KPIs regarding 1595 devices.
(CSV)
