# Supplementary figures and images for: Generative adversarial networks for generating synthetic features for Wi-Fi signal quality
Source: PLoS One. 2021 Nov 23;16(11):e0260308. doi: 10.1371/journal.pone.0260308 (PMC8610258; doi:10.1371/journal.pone.0260308)

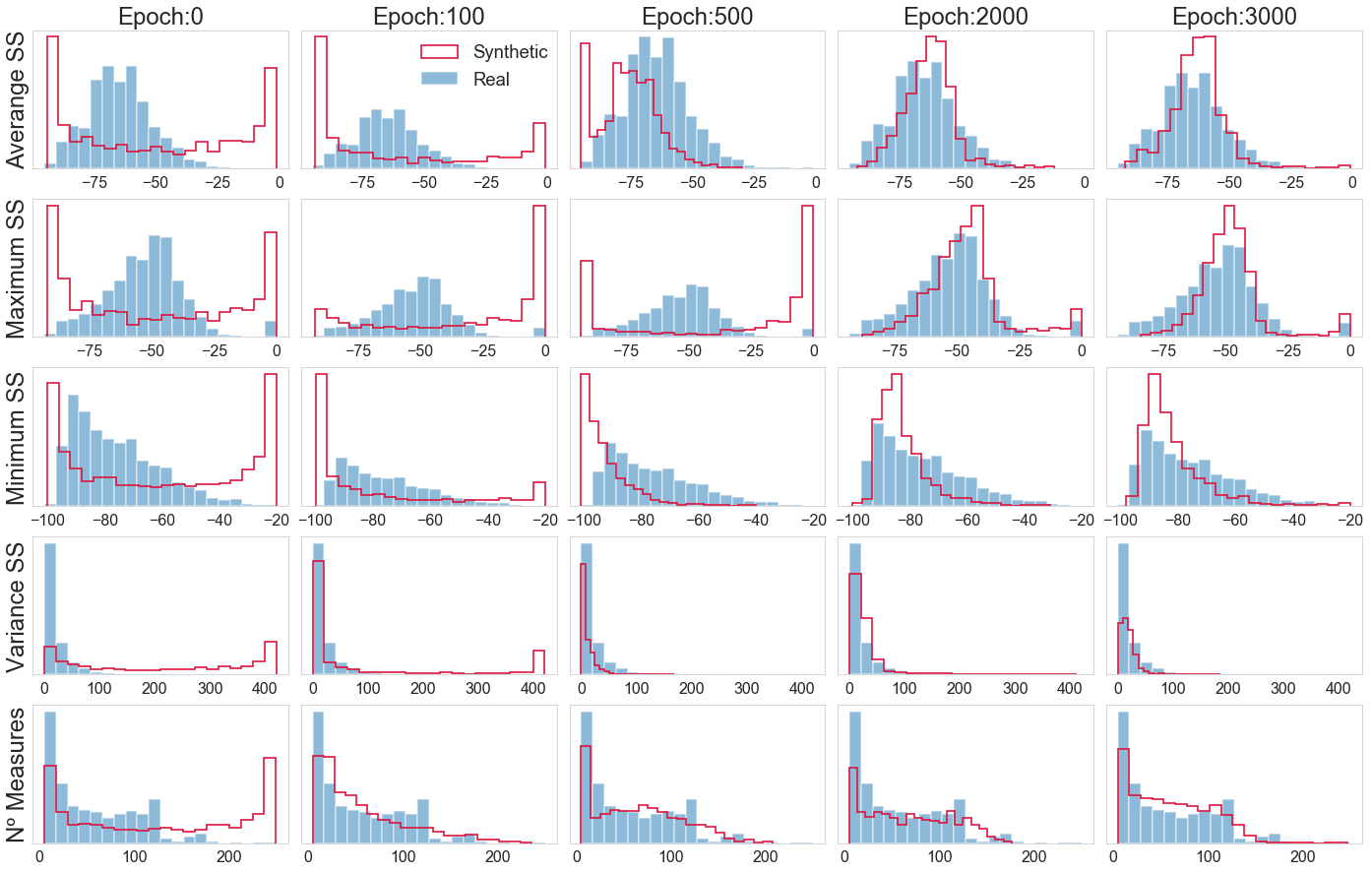

Supplement: S1 Fig — (TIF) [file pone.0260308.s001.tif]

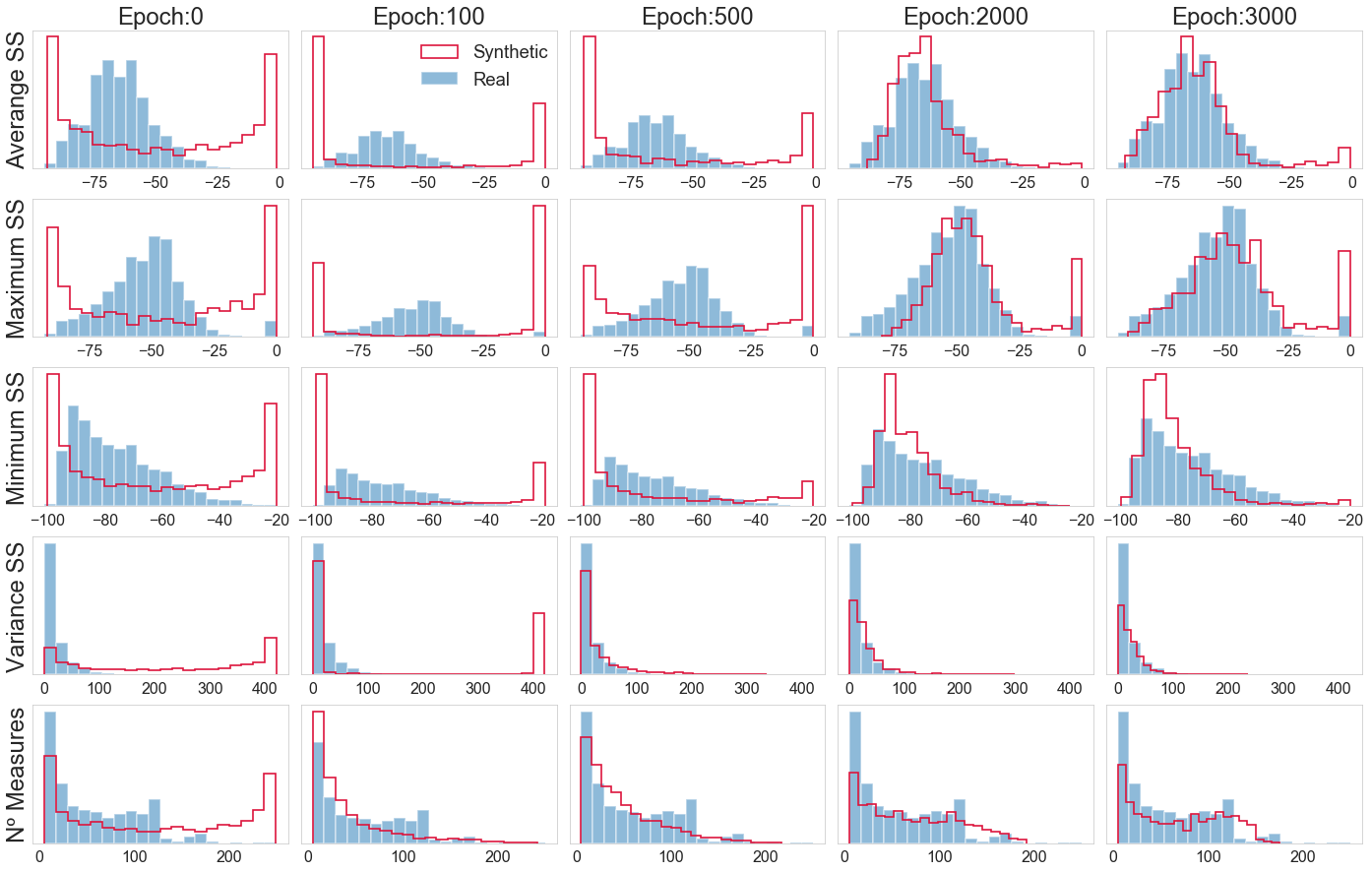

Supplement: S2 Fig — (TIF) [file pone.0260308.s002.tif]

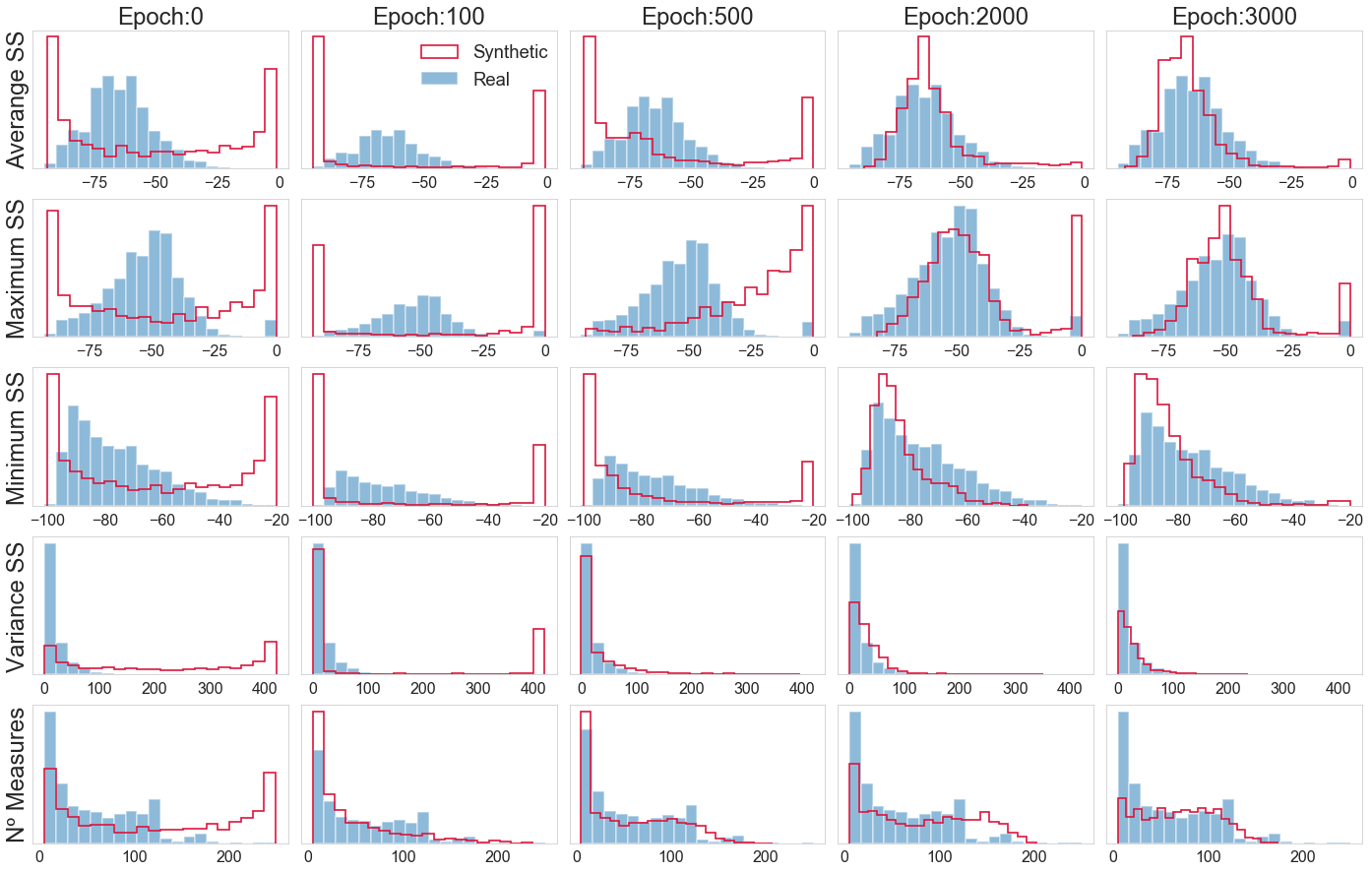

Supplement: S3 Fig — (TIF) [file pone.0260308.s003.tif]

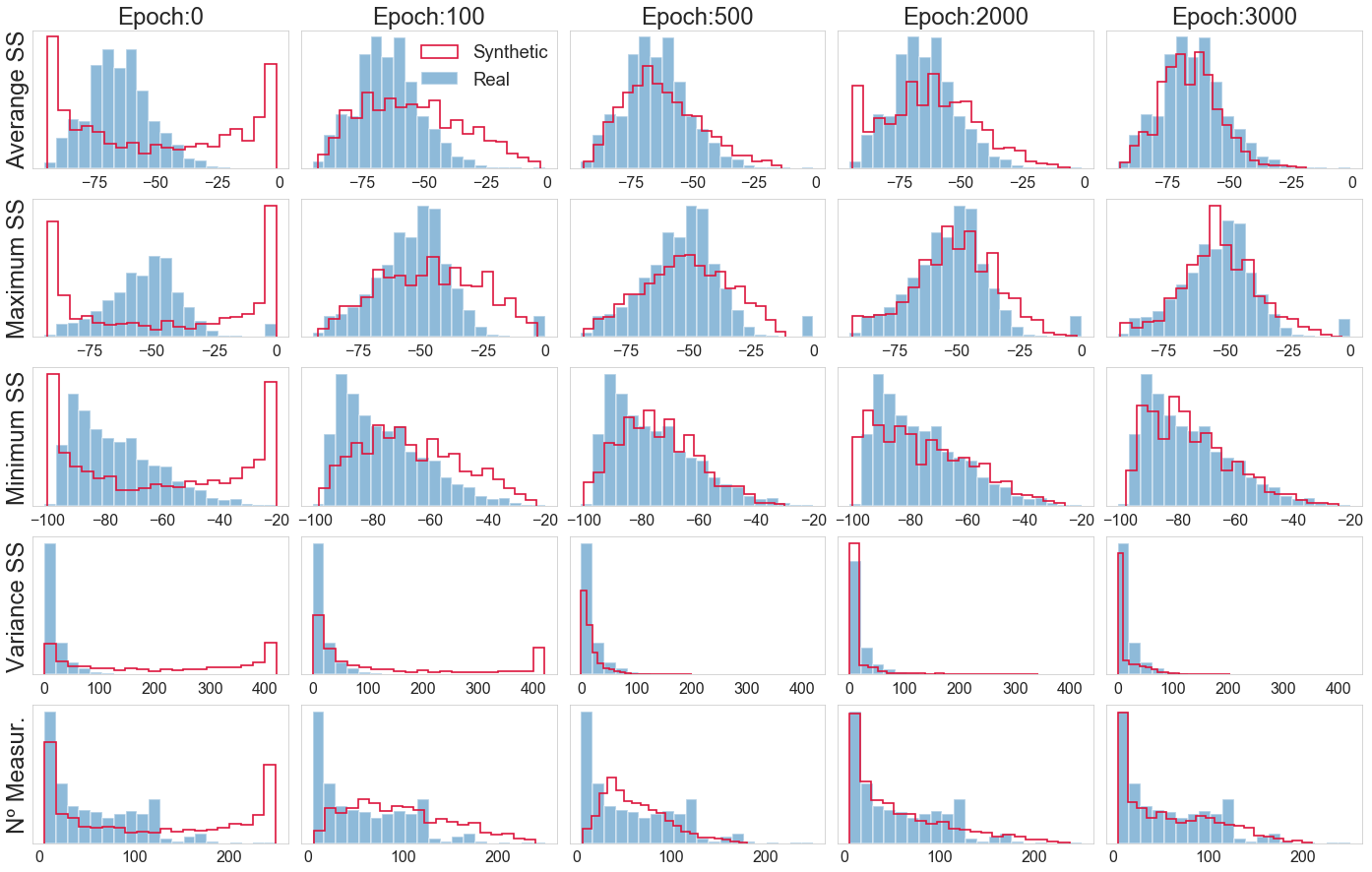

Supplement: S4 Fig — (TIF) [file pone.0260308.s004.tif]

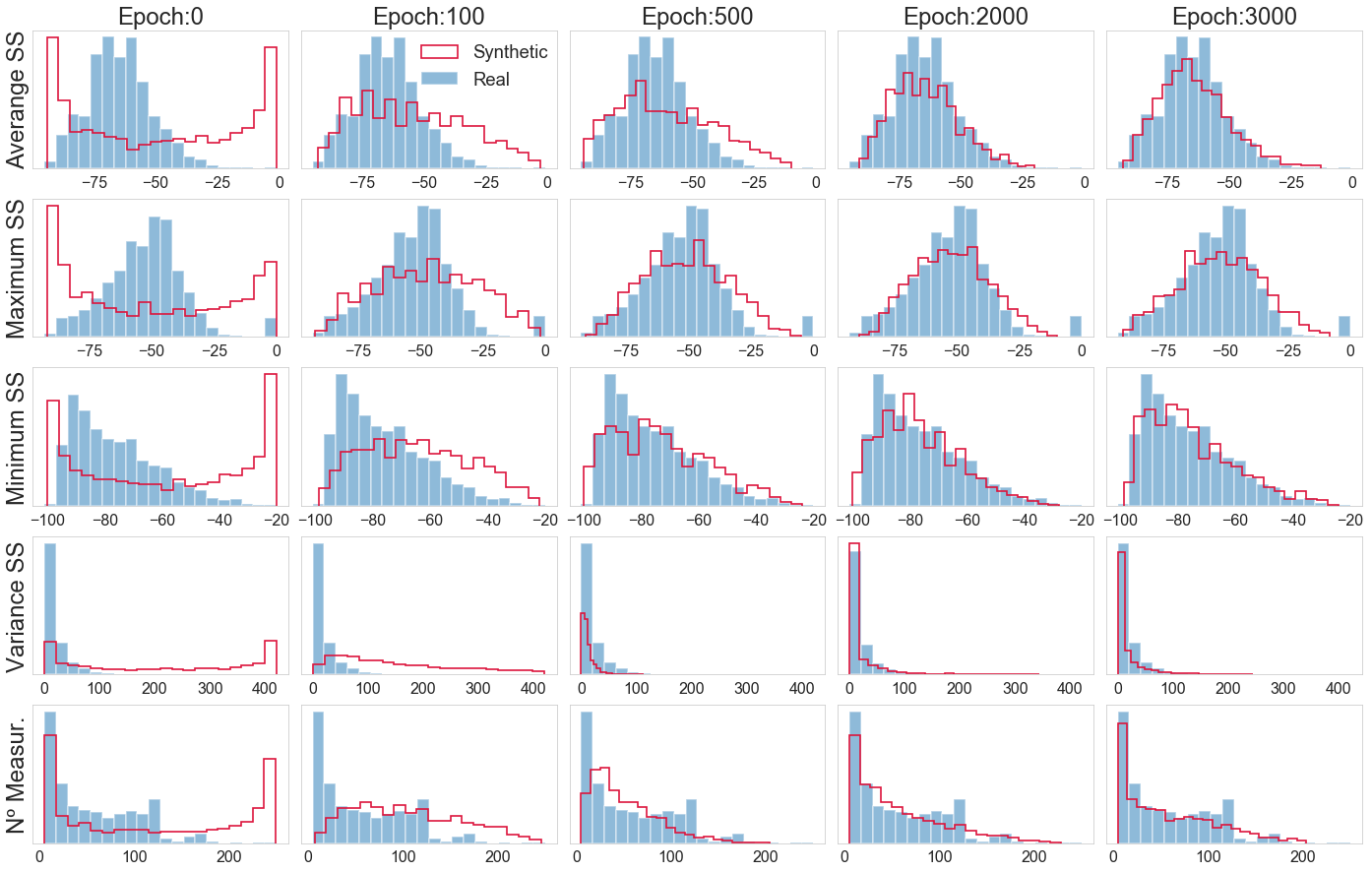

Supplement: S5 Fig — (TIF) [file pone.0260308.s005.tif]

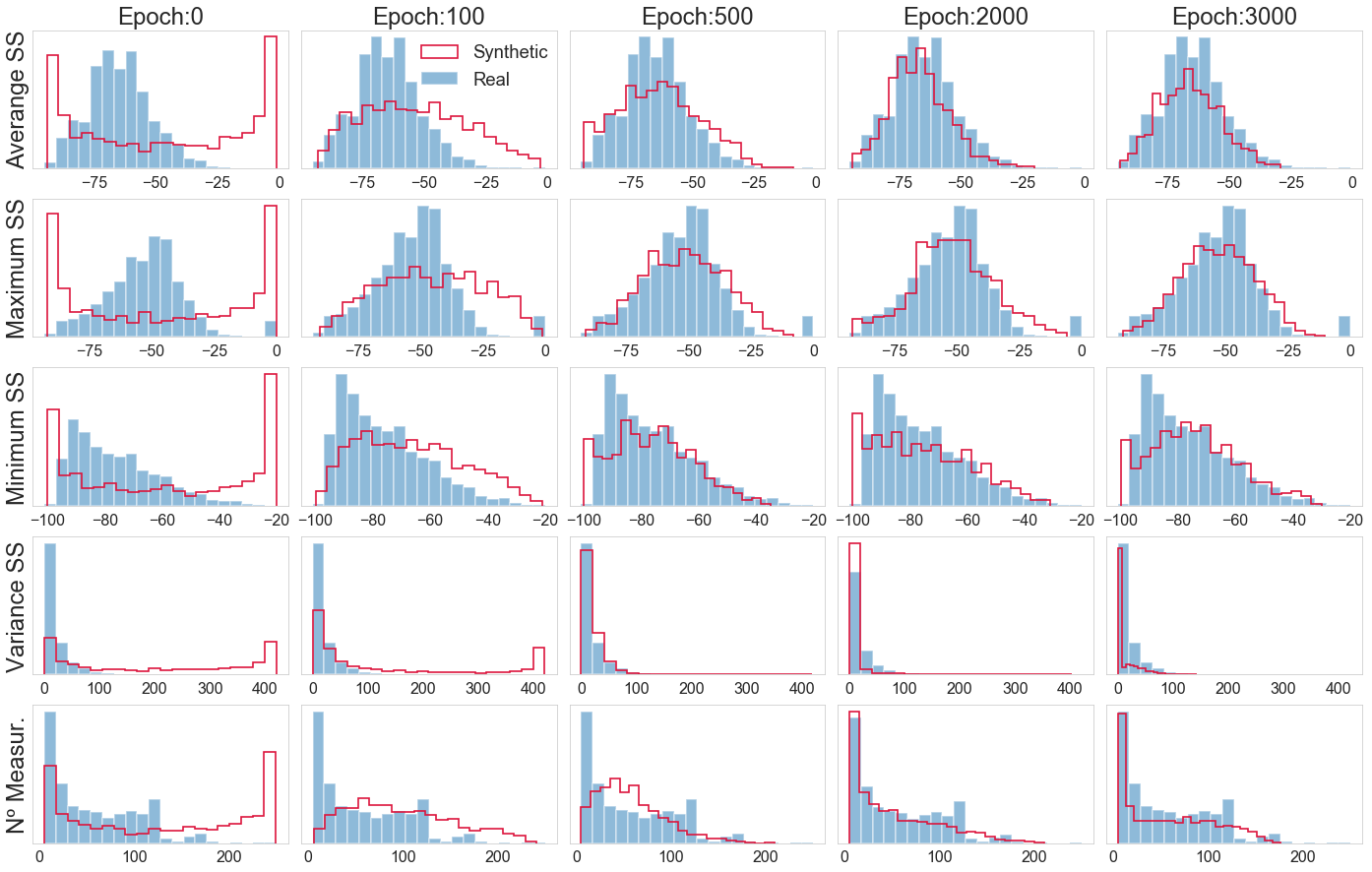

Supplement: S6 Fig — (TIF) [file pone.0260308.s006.tif]
